# TARPγ2 Is Required for Normal AMPA Receptor Expression and Function in Direction-Selective Circuits of the Mammalian Retina

**DOI:** 10.1523/ENEURO.0158-23.2023

**Published:** 2023-08-10

**Authors:** Todd Stincic, Jacqueline Gayet-Primo, W. Rowland Taylor, Teresa Puthussery

**Affiliations:** 1 Herbert Wertheim School of Optometry and Vision Science, University of California, Berkeley, Berkeley, CA 94720; 2 Helen Wills Neuroscience Institute, University of California, Berkeley, Berkeley, CA 94720; 3 Casey Eye Institute, Oregon Health and Science University, Portland, OR 97239

**Keywords:** AMPA, ganglion cells, retina, starburst amacrine cells, TARP

## Abstract

AMPA receptors (AMPARs) are the major mediators of fast excitatory neurotransmission in the retina as in other parts of the brain. In most neurons, the synaptic targeting, pharmacology, and function of AMPARs are influenced by auxiliary subunits including the transmembrane AMPA receptor regulatory proteins (TARPs). However, it is unclear which TARP subunits are present at retinal synapses and how they influence receptor localization and function. Here, we show that TARPɣ2 (stargazin) is associated with AMPARs in the synaptic layers of the mouse, rabbit, macaque, and human retina. In most species, TARPɣ2 expression was high where starburst amacrine cells (SACs) ramify and transcriptomic analyses suggest correspondingly high gene expression in mouse and human SACs. Synaptic expression of GluA2, GluA3, and GluA4 was significantly reduced in a mouse mutant lacking TARPɣ2 expression (stargazer mouse; *stg*), whereas GluA1 levels were unaffected. AMPAR-mediated light-evoked EPSCs in ON-SACs from *stg* mice were ∼30% smaller compared with heterozygous littermates. There was also loss of a transient ON pathway-driven GABAergic input to ON-SACs in *stg* mutants. Direction-selective ganglion cells in the *stg* mouse showed normal directional tuning, but their surround inhibition and thus spatial tuning was reduced. Our results indicate that TARPɣ2 is required for normal synaptic expression of GluA2, GluA3, and GluA4 in the inner retina. The presence of residual AMPAR expression in the stargazer mutant suggests that other TARP subunits may compensate in the absence of TARPɣ2.

## Significance Statement

Transmembrane AMPA receptor (AMPAR) regulatory proteins (TARPs) are molecules that regulate the targeting and functions of AMPA receptors, which are essential for transmitting signals between neurons in the retina and brain. Here, we identify a specific TARP subunit that plays a role in motion-sensitive circuits of the retina. Our results shed light on how AMPAR auxiliary subunit interactions influence neural signaling in the retina.

## Introduction

AMPA receptors (AMPARs) are the major mediators of fast excitatory neurotransmission in the retina and the brain. Four subunits, GluA1–GluA4, combine as homotetrameric or heterotetrameric assemblies to form functional ion channels (Traynelis et al., 2010; Hansen et al., 2021). The functional properties of AMPARs depend on subunit composition as well as interactions with a variety of auxiliary subunits, including the transmembrane AMPA receptor regulatory proteins (TARPs). TARPs are critical for surface expression and synaptic targeting of AMPARs and can markedly alter receptor pharmacology, gating properties and single-channel conductance (Jacobi and von Engelhardt, 2021; Kamalova and Nakagawa, 2021). There are six TARP isoforms (ɣ2 (stargazin), ɣ3, ɣ4, ɣ5, ɣ7, ɣ8) that differ in their relative abundance across different brain regions. It is not yet known which TARPs are present in the retina or which synapses they are associated with.

TARPs were initially discovered in mice harboring a spontaneous mutation that effectively silenced ɣ2 gene expression (Noebels et al., 1990). These mice exhibit absence epilepsy, gait ataxia and episodic upward head movements, and thus were dubbed “*stargazer*” mice. The ataxic phenotype has been linked to the absence of synaptic AMPARs in cerebellar granule cells (Hashimoto et al., 1999; Chen et al., 2000). Indeed, TARPs are known to play a role in trafficking AMPARs to the synapse and selective knock-out of TARP subunits can reduce the number, or alter the subunit composition of, synaptic AMPARs (Tomita et al., 2005; Bats et al., 2013). In some brain regions, multiple TARP isoforms must be eliminated before an overt phenotype is apparent, suggesting an inherent functional redundancy (Menuz et al., 2008).

AMPARs are critical for excitatory neurotransmission in the outer and inner retina. In the outer retina, AMPARs mediate synaptic transmission between photoreceptors and horizontal cells, and, in some species, from photoreceptors to certain OFF cone bipolar cells types (DeVries, 2000; Haverkamp et al., 2001; Puller et al., 2013; Puthussery et al., 2014; Ichinose and Hellmer, 2016). All four AMPAR subunits are expressed in the inner retina, where they localize to dyad synapses between bipolar cells and their postsynaptic amacrine and ganglion cell partners (Brandstätter et al., 1998; Grünert et al., 2002).

Here, we show that the prototypic TARP, TARPɣ2 (stargazin, *stg*), is expressed in the synaptic layers of the retina and shows a highly conserved pattern of localization across mammalian species. TARPɣ2 is relatively enriched in dendrites of OFF and ON starburst amacrine cells (SACs). Quantitative analysis of AMPAR expression in the *stargazer* mutant mouse revealed a role for TARPɣ2 in maintaining normal AMPARs density and synaptic currents at inner retinal synapses.

## Materials and Methods

### Animal use and procedures

All animal procedures were conducted in accordance with the National Institutes of Health guidelines for animal use and a protocol approved by the Institutional Animal Care and Use Committee at Oregon Health & Science University (OHSU). Experiments were performed in adult mice (more than six weeks) of either sex that were homozygous for a spontaneous mutation in the *Cacng2* gene [stargazer (*stg*), JAX strain: 001756, B6C3Fe *a*/*a*-*Cacng2^stg^*/J] or in heterozygous (*het*) or wild-type (*wt*) littermates as indicated. Mice were bred initially from heterozygous breeders obtained from Jackson Labs. The colony was maintained by crossing *stg* homozygotes from the F2 generation to a hybrid *wt* strain that is congenic for the *wt* allele of *Pde6b* [JAX strain: 003647 (C57BL/6JEiJ x C3Sn.BLiA-*Pde6b^+^*/DnJ)F1]. This F1 hybrid is useful for maintaining fragile mutations on a mixed background without the confounding retinal degeneration normally found in C3H strains. *Stg* mutant mice were identifiable by their ataxic phenotype, whereas *wt* and *het* littermates were phenotypically normal. Animals had *ad libitum* access to food and water and were kept on a 12/12 h light/dark cycle.

### Tissue preparation and maintenance

Mice were dark-adapted for at least 1 h before euthanasia and tissue dissection. Mice were deeply anesthetized by intraperitoneal injection of sodium pentobarbital (0.25 ml, 50 mg/ml) and euthanized by cervical dislocation before enucleation. The anterior eye and vitreous were removed and posterior eyecups were processed for immunohistochemistry. For electrophysiological experiments, posterior eyecups were transferred to carbogenated (95% O_2_/5% CO_2_) Ames’ medium for further dissection. Rabbit tissues were obtained from eyes used for unrelated experiments. Anonymized human tissue samples were obtained from eyes enucleated for management of orbital tumors. Tissue use was reviewed by the institutional review board and deemed nonhuman subjects research. Primate eyes were obtained immediately postmortem from animals euthanized in the course of unrelated experiments.

### Antibodies, immunohistochemistry, imaging, and image analysis

The primary antibodies used in this study are detailed in Extended Data Table 1-1. For immunohistochemistry, eyecups were fixed for 5 min in 2–4% paraformaldehyde in 0.1 m phosphate buffer at 25°C. This light fixation approach was necessary to preserve the antigenicity of the GluA and TARP epitopes. After fixation, eyecups were washed in PBS, cryoprotected in graded sucrose solutions (10%, 20%, 30%), embedded in Cryo-Gel (Leica Biosystems), vertically sectioned at 14 μm and stored at −20°C until further use. Cryostat sections were blocked for 1 h in a buffer containing 10% normal horse serum (NHS), 1% Tx-100, 0.025% NaN_3_ in PBS (pH 7.4). Primary antibodies were diluted in 3% NHS, 1% Tx-100, 0.025% NaN_3_ in PBS (pH 7.4) and applied to sections for ∼18 h at 25°C. Secondary antibodies were raised in donkey and conjugated to Alexa Fluor 488 or Alexa Fluor 594. Secondary antibodies were diluted in 3% NHS, 0.025% NaN_3_ in PBS and applied for 1 h at 25°C. Sections were mounted with a Mowiol-based anti-fade medium. Antibody dilutions were titrated for quantitative analysis.

10.1523/ENEURO.0158-23.2023.t1-1 Extended Data Table 1-1 Summary of primary antibodies used in this study. Download Table 1, DOC file

Confocal images were acquired on an Olympus FV1000 confocal microscope with a 60×/1.4 N.A. objective. For GluA, PSD95 and GABA_A_R quantification, experiments were conducted in triplicate (three retinal sections) for each animal (*N* = 6 animals for each genotype) and two image stacks were acquired from each retinal section. Z-stacks of five slices were acquired at the optimal z-interval (0.38 μm) and three out of five sections were maximally projected for intensity profile analysis. All samples for image quantification were processed for immunohistochemistry in parallel under identical conditions and confocal acquisition parameters (magnification, zoom, pixel dwell time, laser power, gain, offset, z-slice thickness, and z-interval) were first adjusted to wild-type samples then kept constant for imaging *stg* samples to permit quantitative comparisons. Care was taken during acquisition to ensure the brightest focal plane was imaged in each sample (to avoid variations in intensity due to antibody penetration) and laser power and PMT gain were adjusted to prevent image saturation.

For the quantitative analysis of immunostaining in the inner plexiform layer, z-stacks were maximally projected and average intensity (mean gray value) was measured as a function of retinal depth from rectangular regions of interest spanning the entire inner plexiform layer (IPL) and adjacent inner nuclear and ganglion cell layers. IPL depth was normalized across different samples using the dendritic stratification of choline acetyltransferase (ChAT)-expressing starburst amacrine cells (SACs) for reference. This permitted averaging of intensity profiles from different retinal regions that had slightly different IPL thicknesses. To determine the depth of stratification of the SACs, we first measured the mean ChAT fluorescence intensity as a function of IPL depth and identified local peaks in the intensity profiles using the FindPeak operation in Igor Pro, which searches for local maxima by analyzing the smoothed first and second derivatives of the input data. For samples where calretinin was used as a reference marker, the peaks of the inner and outermost bands were used for reference since these bands correspond to the ON and OFF SACs. The x-scaling of each intensity profile was then adjusted to align the ChAT or calretinin peaks across all samples and to set the OFF and ON-ChAT bands to 28% and 63% of the IPL depth respectively (Helmstaedter et al., 2013; Li et al., 2016). The same x-scaling factors were then applied to intensity profiles of the receptor subunits, which were imaged in a separate channel of the same images. For each animal, the average intensity profile was obtained by averaging profiles from two images from three separate sections (i.e., six images in total) to account for any variability in staining intensity across different regions of the section or slide. The resulting fluorescence intensity profiles from *stg* retinas were normalized to the peak intensity measurements from *wt* retinas imaged in the same session with identical acquisition parameters to account for any absolute differences in intensity that could have been imposed by the confocal system across sessions. Lasers were warmed up for at least 30 min before beginning acquisition. Image analysis was performed using FIJI and custom routines in Igor Pro 7/8.

### Single-cell RNA-sequencing analysis

For transcriptomic analysis, we mined existing scRNA-seq datasets from human (Accession: GEO:GSE148077; Yan et al., 2020b) and mouse retina (Accession: GEO:GSE149715; Yan et al., 2020a). Cell cluster assignments were as reported in the original publications. Dot-plot visualizations were generated using the Broad Institute Single-Cell Portal (https://singlecell.broadinstitute.org/single_cell), where dot size indicates the proportion of cells in the cluster that expressed the gene and dot color indicates the relative gene expression level for each row.

### Electrophysiology and light stimulation

Retinas were isolated from posterior eye cups in carbogenated Ames’ medium under infrared illumination (850 nm). Radial cuts were made in whole or half retinas and pieces were mounted on an aluminum oxide membrane (Whatman Anodiscs) and stabilized with a nylon “harp.” Warmed 32–34°C Ames’ medium was perfused through the chamber at a flow rate of 3–4 ml/min.

Extracellular and patch electrodes were pulled from borosilicate glass to a final resistance of 3–9 MΩ. For loose cell-attached recordings, electrodes were filled with Ames’ medium. For patch-clamp recordings, electrodes contained (in mm): 128 Cs-methanesulfonate, 6 CsCl, 10 Na-HEPES, 1 EGTA, 2 Mg-ATP, 1 Na-GTP, 5 phospho-creatine, 3 QX-314, and 0.1 spermine adjusted to pH 7.4 with CsOH. All reagents were obtained from Sigma-Aldrich unless otherwise indicated. Cesium was used in place of potassium to block voltage-gated potassium currents and thereby improve voltage clamp at positive potentials. QX-314 was included to block voltage-gated sodium channels and abolished all spiking activity within 1–2 min of establishing the whole-cell configuration. A liquid junction potential of −10 mV was subtracted from all voltages.

We targeted cells for recordings under infrared illumination (850 nm) using Dodt contrast optics. To target SACs, small, round somas ∼8–10 μm were first loose patched to confirm a lack of spike activity before whole-cell recordings were made. To further confirm SAC identity, fluorescent dye (Alexa 488 hydrazide, Invitrogen) was added to the intracellular solution to confirm cell morphology at the end of the recordings. An effort was made to record from SACs closer to the optic nerve since the smaller size of the dendritic arbors improved the voltage-clamp. To target direction-selective ganglion cells (DSGCs), larger, elongated somas were loose patched and probed with a 150 μm spot. Cells with ON-OFF responses were then probed with drifting bar stimuli to test for direction selectivity.

Spot stimuli were centered on the receptive field, and intensity was increased (bright spot) or decreased (dark spot) from the background level. All stimuli were generated on CRT computer monitors at refresh rates of 60 or 85 Hz. The stimuli were projected through the microscope and focused onto the photoreceptor outer segments, through an Olympus 20×/0.95 N.A. water-immersion objective. Contrast was defined as *C* = 100(*L*_max_ − *L*_min_)/(*L*_max_ + *L*_min_), where *L*_max_ and *L*_min_ are the maximum and minimum intensities of the stimulus, respectively. The standard stimulus was a circular spot centered on the receptive field of the cell presented on a steady background of ∼10^5^ photons/μm^2^/s, well above the scotopic range.

The background light intensity (LBACK) was set to 150 μW/m^2^ at the retinal surface. The stimulus light intensity (LSTIM) was set to 30 μW/m^2^ for dark stimuli and to 270 μW/m^2^ for bright stimuli. Thus, the percentage stimulus contrast, defined as C = 100×(L_STIM_–L_BACK_)/L_BACK_, ranged from –80% to +80%.

### Electrophysiological analysis

Analysis was performed using custom procedures in IgorPro 8/9 (Wavemetrics). The preferred-null axis of ON-OFF DSGCs was measured by counting the number of spikes elicited by drifting dark bars across the receptive fields of the cells at 1 mm/s in 12 different directions (30° intervals). Peristimulus spike-time histograms (PSTHs) were generated by accumulating spikes from 3 trials in each direction. The average number of spikes per trial for each angle was estimated by integrating the respective PSTHs during the ON and OFF responses. The vectors, one for each angle, were summed and the preferred direction calculated as the angle of the resultant vector. A direction-selectivity index (DSI) was calculated as *(R_pref_ – R_null_)/(R_pref_ + R_null_)*, where *R* is the response amplitude, and “pref” and “null” refer to stimuli in the preferred and null directions.

Conductance was calculated using previously described methods (Taylor and Vaney, 2002). Briefly, light evoked synaptic currents are assumed to result from the sum of linear excitatory and inhibitory synaptic conductances. Synaptic conductance was estimated from I-V relations of net light-evoked currents measured from light-responses during voltage steps from −90 to +50 mV. Conductances were estimated from least-square fits to the I-V relations, assuming reversal potentials for excitation and inhibition of 0 mV and −69 mV, respectively. I-V relations were constructed and fit at 10-ms intervals to reveal the time course of the synaptic conductance.

Spontaneous EPSCs (sEPSCs) were quantified as follows. Intervals of current recordings were first differentiated, which effectively removed the slow fluctuations and emphasized larger, rapidly-activating events. Individual event times were then detected by thresholding the derivative traces at 5 SDs of the noise. Sections of the record were then excised around each event and averaged together to produce the average sEPSCs. For the *het* data, 38 s of recording were analyzed from five ON-SACs in four animals. For the *stg* data, 46 s were analyzed from five ON-SACs in three animals.

### Statistical analysis

Unless noted otherwise, data are shown as mean ± SD. Statistical comparisons were made using two-tailed, paired or unpaired *t* tests or in the case of unpaired nonparametric data with the Mann–Whitney *U* test using an α level of 0.05. Normality testing was done with the Shapiro–Wilk test. Welch’s correction was applied where necessary to correct for unequal variances. Repeated measures ANOVA was used to analyze results in Figures 10 and 11. The Bonferroni correction was made to account for the multiple comparisons made for data in Figure 4 (adjusted α level of 0.05). Statistical comparisons were made using Igor Pro 9 (Wavemetrics) or Prism 9 (GraphPad). A summary of all statistical comparisons is included in Extended Data Table 2-1.

10.1523/ENEURO.0158-23.2023.t2-1 Extended Data Table 2-1 Summary of statistical tests used in this study. Download Table 2, DOC file

## Results

### TARPɣ2 is expressed in the synaptic layers of the retina

**Figure 1.**
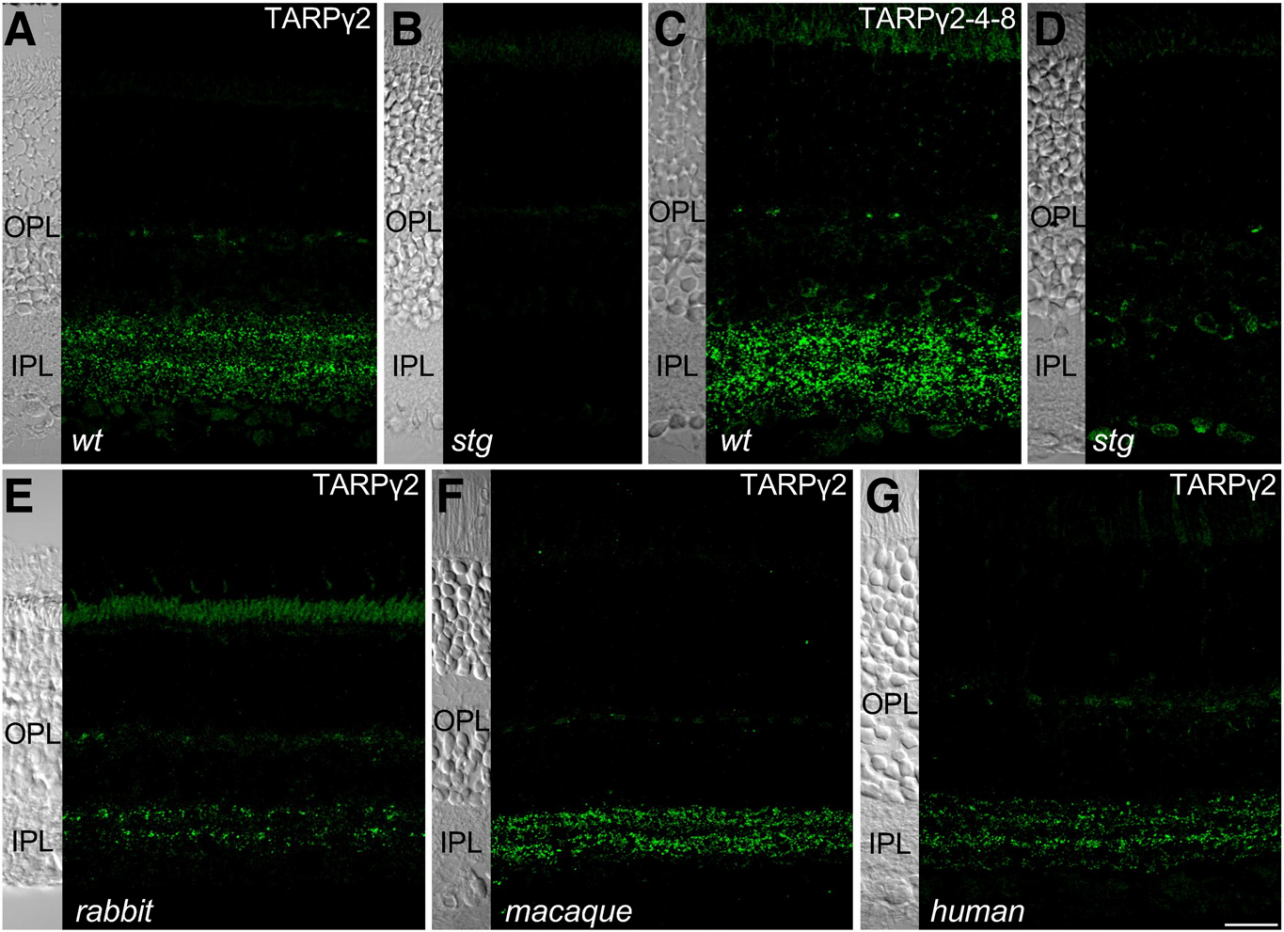
TARPγ2 localization is conserved across mammalian species. ***A***, ***B***, Synaptic localization of TARPγ2 (***A***, ***B***) and TARPγ2/4/8 (***C***, ***D***) in *wt* and *stg* mutant mouse retina. Note that both TARPγ2 and TARPγ2/4/8 were absent in the IPL of the *stg* mutant mouse. ***E–G***, Localization of TARPγ2 primarily in the OPL and IPL of the rabbit (***E***), macaque (***F***), and human (***G***) retina. Left side of each panel shows a transmitted light image of the same field of view used to visualize retinal layers. Scale bar in ***G* **=** **20 μm and applies to all panels.

Our first objective was to determine the identity and localization of the TARP subunits in mammalian retina. To this end, we used two antibodies, one that recognizes TARPɣ2, and another that recognizes an epitope common to TARPɣ2, TARPɣ4, and TARPɣ8 (TARPɣ2/4/8). TARPɣ2 showed punctate labeling in both the outer and inner plexiform layers (IPLs) in wild-type (*wt*) mouse retina (Fig. 1*A*). This staining was absent in stargazer mutant (*stg*) mouse retinas (Fig. 1*B*). Labeling for TARPɣ2/4/8 was generally stronger but synaptic staining was absent in the *stg* mouse (Fig. 1*C*,*D*) suggesting that ɣ4 and ɣ8 subunits are not expressed at significant levels. To test whether TARP expression was conserved in other mammalian species, we immunolabeled for TARPɣ2 in rabbit, macaque, and human retina (Fig. 1*E–G*). In all species, the expression of TARPɣ2 in the outer plexiform layer (OPL) was generally weak, although the presence of intermittent clusters in this region suggests staining associated with cone pedicles. As in the mouse, strong staining was observed in the IPL. Overall, the similarity in staining pattern across species suggests conserved expression of TARPɣ2.

**Figure 2.**
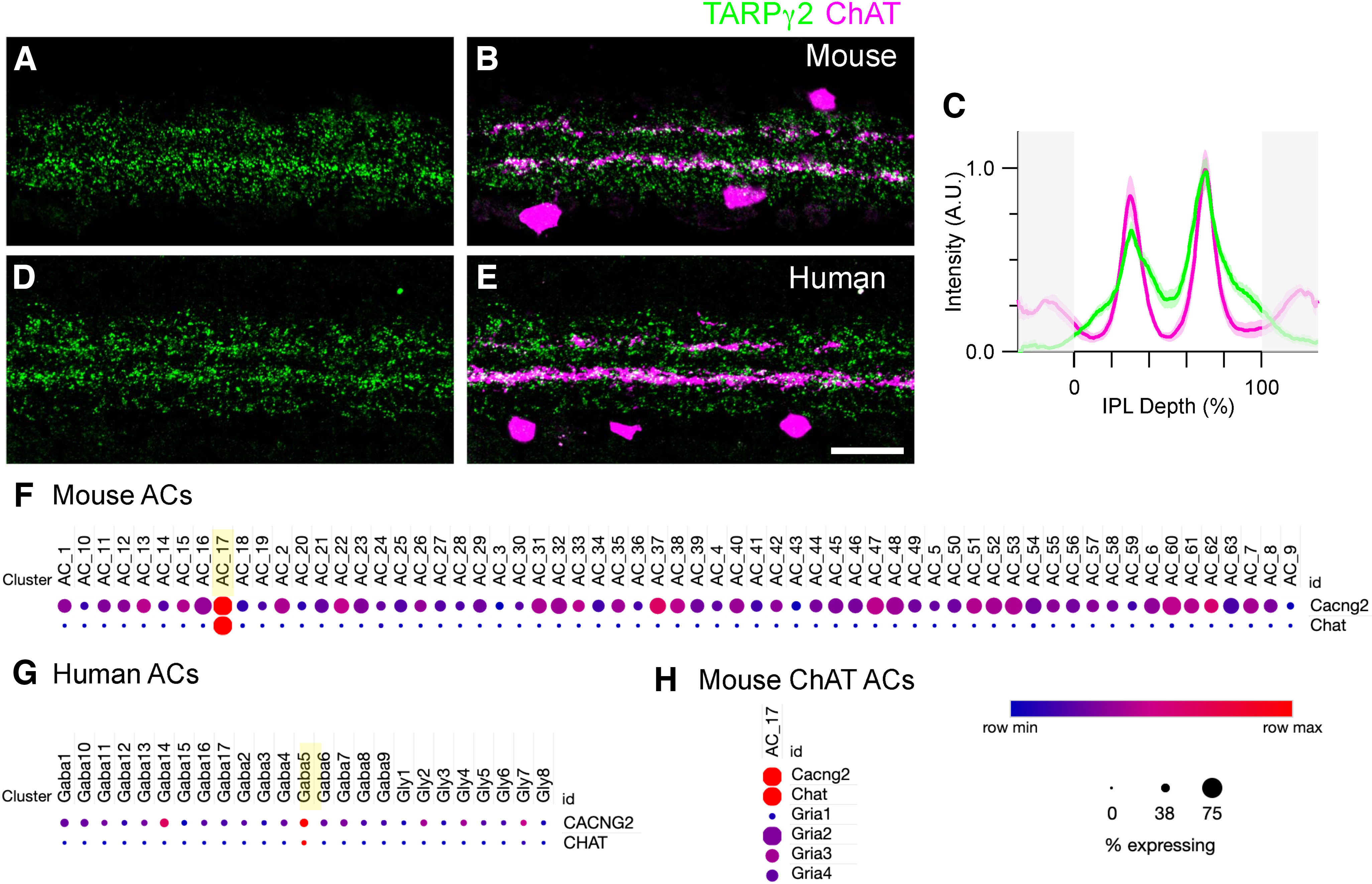
TARPγ2 expression is concentrated in SACs. ***A***, ***B***, Localization of TARPγ2 and ChAT in the IPL of the wild-type mouse retina. ***C***, Average normalized fluorescence intensity profiles of TARPγ2 and ChAT in the mouse IPL (*N* = 6 mice). The peaks of TARPγ2 staining align with the dendrites of the OFF and ON-ChAT bands. Shading represents ± 1 SEM 0% is the outer and 100% is the inner border of the IPL, respectively. ***D***, ***E***, Localization of TARPγ2 and ChAT in the IPL of a human retina shows a similar pattern to mouse. Scale bar in ***E*** = 20 μm and applies to ***A***,***B***,***D***,***E***. ***F***, Relative expression of *Cacng2* (TARPγ2) transcript in different mouse amacrine cell types. The ChAT-expressing amacrine cells (SACs, AC_17) show relatively higher levels of *Cacng2* than other amacrine cell types. Raw data from Yan et al. (2020a,b). ***G***, Relative expression of *Cacng2* transcript across different human amacrine cell types shows higher levels of *CACNG2* expression in SACs (Gaba5). ***H***, Expression of AMPAR subunits in SACs (AC_17) relative to other mouse amacrine cell types (data not shown). Note that *Gria1* levels are low in SACs, whereas transcript levels of *Gria2*, *Gria3*, and *Gria4* are higher. Dot size indicates % expressing, dot color indicates relative expression level across rows.

### TARPɣ2 is enriched in starburst amacrine cells

Although TARPɣ2 was localized throughout the IPL, we noted two more prominent “bands” of immunoreactive puncta, that were particularly apparent in mouse, rabbit, and human retina. To determine the stratification level of these bands, we co-labeled with an antibody against choline acetyltransferase (ChAT), a marker of OFF-type and ON-type starburst amacrine cells (SACs), which are often used as fiducial markers of stratification in the IPL. The OFF and ON-SAC dendrites stratify at ∼28% and ∼63% depth of the IPL, respectively (where 0% is the border of the inner nuclear layer and 100% the border of the ganglion cell layer; Li et al., 2016). The two strongest bands of TARPɣ2 staining were coincident with the OFF and ON-SAC dendrites in mouse retina (Fig. 2*A–C*). The normalized peak intensity of TARPɣ2 was ∼32 + 9.6% (mean + SD) lower in the OFF- compared with the ON ChAT band (Fig. 2*C*, *p* = 0.0039, paired *t* test, *N* = 6 mice). Similar to the mouse, the strongest bands of TARPɣ2 in human retina coincided with the ChAT bands (Fig. 2*D*,*E*). In both species, TARPɣ2 puncta appeared to colocalize with ChAT+ dendrites, suggesting possible expression in SACs. However, direction-selective ganglion cells (DSGCs) co-stratify with SACs and could also express TARPɣ2. To determine which of these neurons expressed TARPɣ2, we mined existing single-cell transcriptomic datasets from mouse and human retina (GEO: GSE149715; GSE149715; Yan et al., 2020a,b). In both species, SACs showed higher expression of *Cacng2*, the gene that encodes TARPɣ2, compared with all other amacrine cell types (Fig. 2*F*,*G*). In mouse, DSGCs also express *Cacng2*, but transcript levels did not differ markedly from other RGC types (data not shown, dataset from (Tran et al., 2019; GEO: GSE133382). Overall, these results suggest that TARPɣ2 is expressed at a variety of amacrine and ganglion cell synapses in the IPL, but that SACs have relatively higher levels of expression.

### TARPɣ2 colocalizes with AMPAR subunits

**Figure 3.**
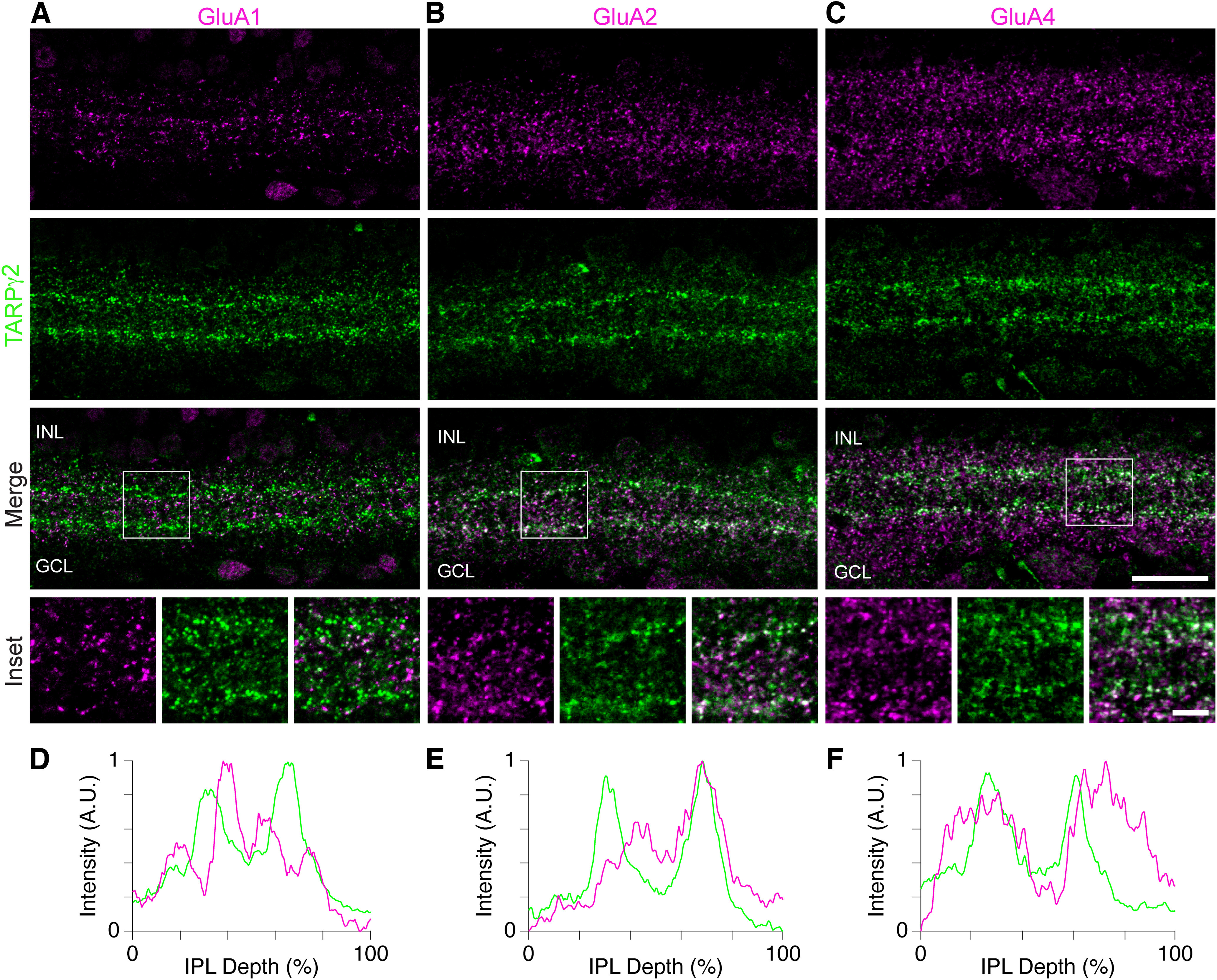
TARPγ2 is associated with specific GluA receptor subunits in mouse retina. ***A–C***, Confocal images of the mouse IPL showing double labeling of GluA1 (***A***), GluA2 (***B***), and GluA4 (***C***) subunits (top panels) with TARPγ2 (second row). Merge of GluA subunits and TARPγ2 in shown in third row. Square ROIs in merged images are shown enlarged below (inset). ***D–F***, Normalized fluorescence intensity profiles of TARPγ2 (green) and GluA1 (***D***), GluA2 (***E***), and GluA4 (***F***) subunits (magenta) from projected confocal image stacks of the IPL. Scale bar in ***C*** merge applies to all main panels = 20 μm. Inset scale bars = 5 μm.

To determine which AMPAR subunits were associated with TARPɣ2 in SACs, we examined the transcript levels of the genes encoding the AMPAR subunits GluA1–GluA4 (*Gria1, Gria2, Gria3, Gria4*) in mouse SACs. Consistent with reports at the protein level in rabbit retina (Firth et al., 2003), *Gria1* is expressed at relatively low levels in SACs compared with other amacrine cell types, whereas *Gria2, Gria3, Gria4* are present at higher levels (Fig. 2*H*). To confirm this result at the protein level, we compared the localization of GluA1, GluA2, and GluA4 with TARPɣ2 (Fig. 3*A–C*). GluA1 showed little colocalization with puncta in the stronger TARPɣ2 bands and the GluA1 intensity profiles appeared to dip where TARPɣ2 intensity was highest. Conversely, GluA2 and GluA4 puncta colocalized with TARPɣ2 at the level of the stronger TARPɣ2 bands (Fig. 3*D–F*). These results suggest that TARPɣ2 associates with specific AMPAR subunits in SACs. We provide further quantitative evidence to support this conclusion in the next section.

**Figure 4.**
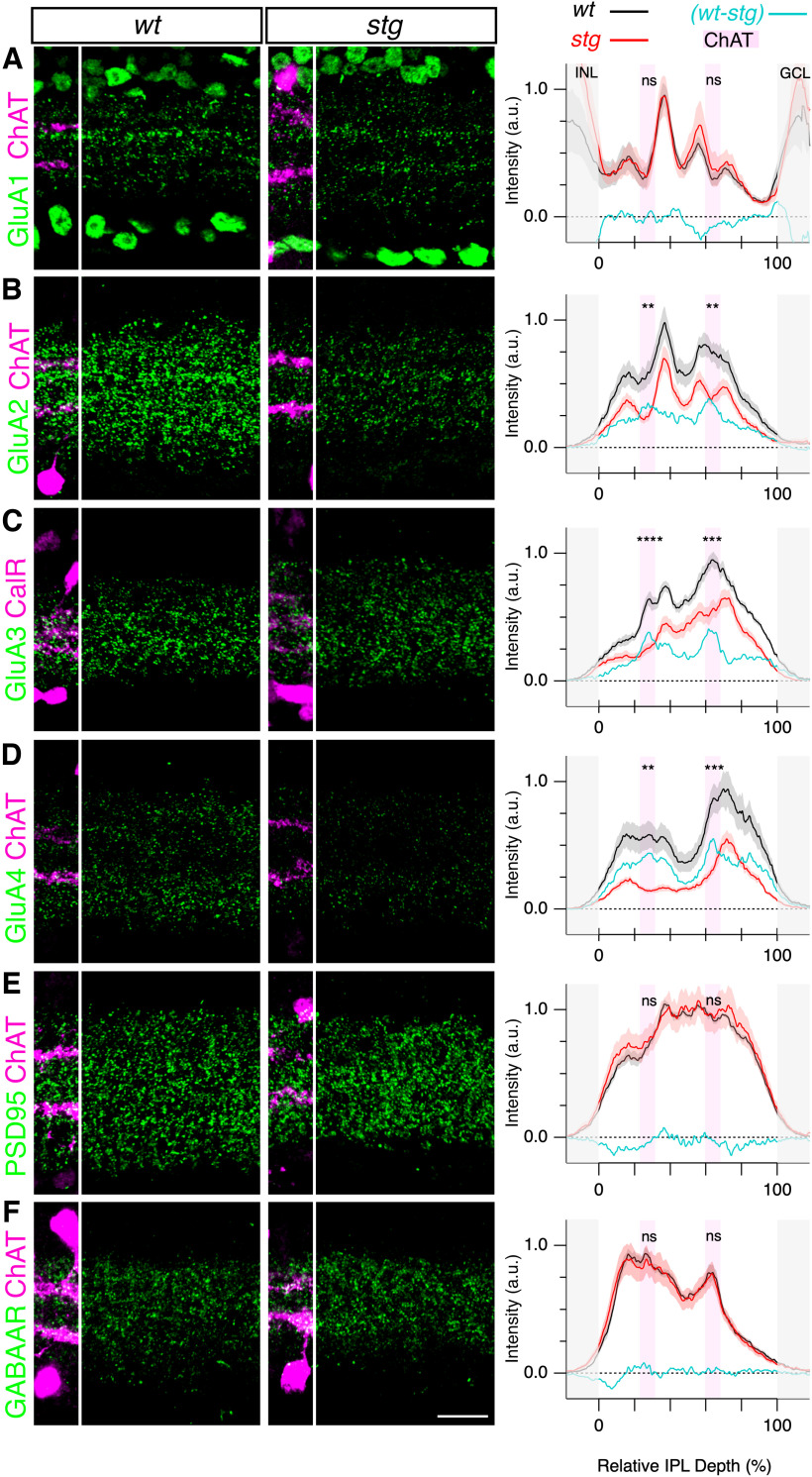
Absence of TARPγ2 reduces expression of specific AMPAR subunits in the mouse inner retina. ***A–D***, Immunolocalization and intensity profiles of GluA subunits (***A–D***), PSD95 (***E***), and GABA_A_R β2-β3 subunit (***F***), in the IPL of *wt* (*left*) and *stg* mutant mouse (center). ChAT or calretinin were used as reference markers in ***A–D*** and are shown in magenta. Right, Normalized average fluorescence intensity profiles from *wt* (black) and stg mice (red). The average values were obtained from six retinas from six independent animals for each genotype. Shading shows ± 1 SEM. Cyan lines are difference plots showing *wt* - *stg*. Pink shading shows position of ChAT bands in ***A***, ***B***, ***D***, and outer CalR bands in ***C***. Statistical comparisons are taken at the position of the OFF and ON ChAT bands using unpaired *t* tests with the Bonferroni correction for multiple comparisons (see also Extended Data Table 2-1). ns = not significant, **p <* *0.01, ***p <* *0.001, ****p <* *0.0001. Scale bar in ***F*** = 20 μm applies to all images.

**Figure 5.**
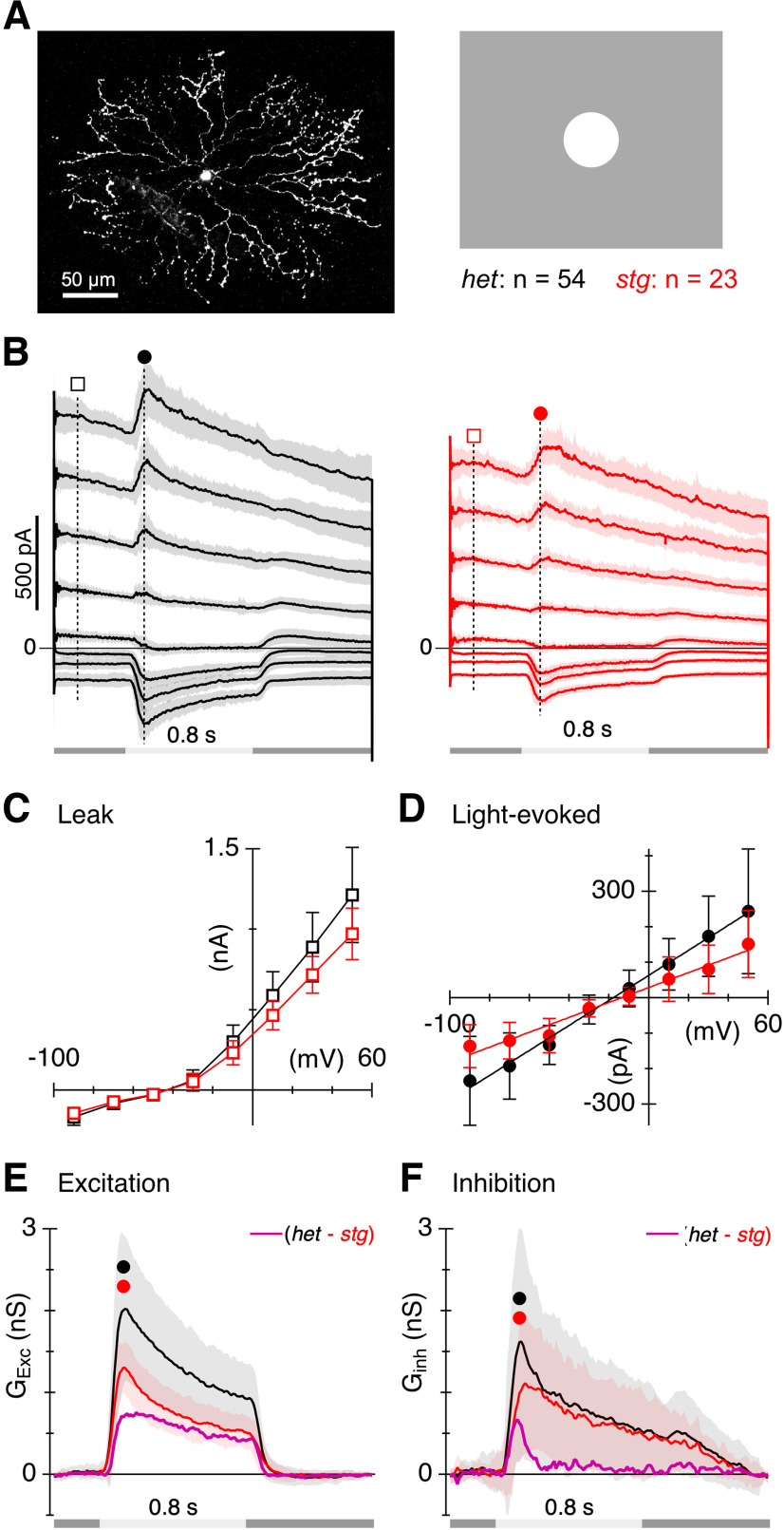
Absence of TARPγ2 reduces excitatory input to ON-SACs. ***A***, Example of an ON-SAC filled with Alexa 488 hydrazide. ***B***, Total membrane current during depolarizing voltage steps to holding potentials between −90 and +60 mV. Timing of a 175 μm diameter positive contrast stimulus spot shown beneath traces. Shading and error bars show ±1 SD ***C***, Average current–voltage relations for the passive membrane conductance measured at the time points shown by the square symbols in ***B***. ***D***, Average net light-evoked EPSC amplitudes measured at the time points indicated by the circle symbols in ***B***. Lines show the linear fits used to calculate the excitatory and inhibitory conductance components shown in ***E***. ***E***–***F***, Excitatory and inhibitory conductances were calculated for the data in ***B***. Circles indicate the time points used to calculate peak conductances. The magenta traces show the difference between the *het* and *stg* traces. These show the net conductance that is lost in the *stg* retinas. Error bars and shading are ±1 s.d.

### TARPɣ2 is required for normal synaptic expression of GluA2, GluA3, and GluA4

In other brain regions, the absence of TARPɣ2 alters the density and synaptic targeting of AMPAR subunits (Chen et al., 2000). To determine whether the absence of TARPɣ2 altered AMPAR expression in the retina, we examined AMPAR expression in the *stg* mutant mouse. Since TARPɣ2 protein expression was highest at the level of the SAC dendrites (“ChAT bands”; Figs. 2, 3), we hypothesized that the absence of TARPɣ2 would have the most impact on the expression of GluA subunits in SACs. Moreover, GluA2 and GluA4 expression should be altered more so than GluA1, since there is little GluA1 expression in SACs (Figs. 2*H*, 3*D*). To test these predictions, we quantified the average fluorescence intensity of each GluA subunit as a function of IPL depth in *wt* and *stg* mutant retinas (Fig. 4, *N* = 6 mice). The absence of TARPɣ2 did not alter dendritic stratification of SACs and there was no obvious change in SAC cell density (data not shown). In *wt* retina, GluA1 varied with IPL depth, with intensity peaks at ∼ 36% and 56% depth of the IPL and two smaller peaks at ∼17.5% and 70% depth (Fig. 4*A*, right panel). GluA1 levels dipped at the level of the ChAT bands consistent with the results shown in Figure 3. The intensity profiles for GluA1 were comparable in the *stg* mutant and *wt* mouse suggesting that TARPɣ2 is not required for normal expression of GluA1 in the inner retina (Fig. 4*A*). In contrast, there was a significant reduction in GluA2, GluA3, and GluA4 signal in *stg* compared with *wt* mice (Fig. 4*B–D*). Difference plots (*wt* – *stg*) showed that although expression was reduced across the entire IPL, the largest reductions in GluA signal intensity were at the level of the OFF-ChAT and ON-ChAT bands (Fig. 4*B–D*, right panels, cyan). As a control, we analyzed the postsynaptic density scaffold protein, PSD95, which interacts with the TARPγ2 PDZ-binding domain to maintain AMPARs at the synapse (Chen et al., 2000; Schnell et al., 2002; Dakoji et al., 2003). PSD95 expression was comparable in *stg* and *wt* retinas, indicating that loss of TARPɣ2 does not impact the overall expression of the postsynaptic scaffold protein in the IPL (Fig. 4*E*). As an additional control, we quantified the GABA_A_R β2, β3 subunit, which is targeted to GABAergic synapses independently of TARPs. As expected, the absence of TARPɣ2 did not alter the intensity profiles of the GABA_A_R subunit (Fig. 4*E*). Together, these results indicate that TARPɣ2 is required for normal inner retinal expression of GluA2, GluA3, and GluA4, but not GluA1. The largest reductions in AMPAR signal intensity were seen at the level of the OFF-SACs and ON-SACs, corresponding to the region of highest TARPɣ2 signal. Given the requirement of TARPɣ2 for normal expression levels of GluA2, GluA3, and GluA4 in SACs, we next tested the functional impact of the loss of TARPɣ2 on the synaptic inputs to these cells.

### Absence of TARPɣ2 reduces excitatory currents in on-SACs

We targeted ON-type SACs, which have somas in the ganglion cell layer and can be identified based on dendritic morphology and confirmed by physiological criteria. Morphology was visualized by filling cells with fluorescent dye during the recordings (Fig. 5*A*). To determine whether loss of TARPɣ2 affected excitatory input to ON-SACs, we recorded responses to light spots covering the center of the receptive field at a range of holding potentials from −90 to +50 mV in 20-mV increments (Fig. 5*B*). The leak-current during voltage-steps was similar in the two groups, at least over the expected physiological operating range between about −60 and −40 mV, indicating that the passive membrane properties were largely unchanged in the mutant (Fig. 5*B*,*C*). However, the peak light-evoked excitatory conductance was ∼36% smaller in the mutant SACs, evident as a reduction in the slope of the synaptic I-V relation (Fig. 5*D*) in the *stg* mutant relative to the *het* control and quantified as a suppression of the peak excitatory synaptic conductance (Fig. 5*E*, *het* control 1.75 ± 0.82 nS, *n* = 54 cells, *stg* 1.12 ± 0.27 nS, *n* = 23 cells, *p* = 2.6 × 10^−6^). The magenta trace in Figure 5*E* shows the excitatory conductance that is lost in the *stg* SACs. Note that this component is more sustained than the excitation seen in the *het* controls, suggesting the loss of a kinetically distinct component of AMPAR-mediated excitatory inputs. The ON-SACs in the *stg* mutant also showed a 38% reduction of the peak inhibitory conductance compared with the *het* control group (Fig. 5*F*, *stg/+* peak = 1.36 ± 1.16 nS, *n* = 54 cells, *stg/stg* peak = 0.85 ± 0.67 nS, *n* = 23 cells, *p* = 0.018), whereas a sustained component of the inhibitory conductance, and the inhibition at the termination of the light flash, were unaffected. The magenta trace in Figure 5*F* shows the inhibitory conductance that is lost in the *stg* SACs.

**Figure 6.**
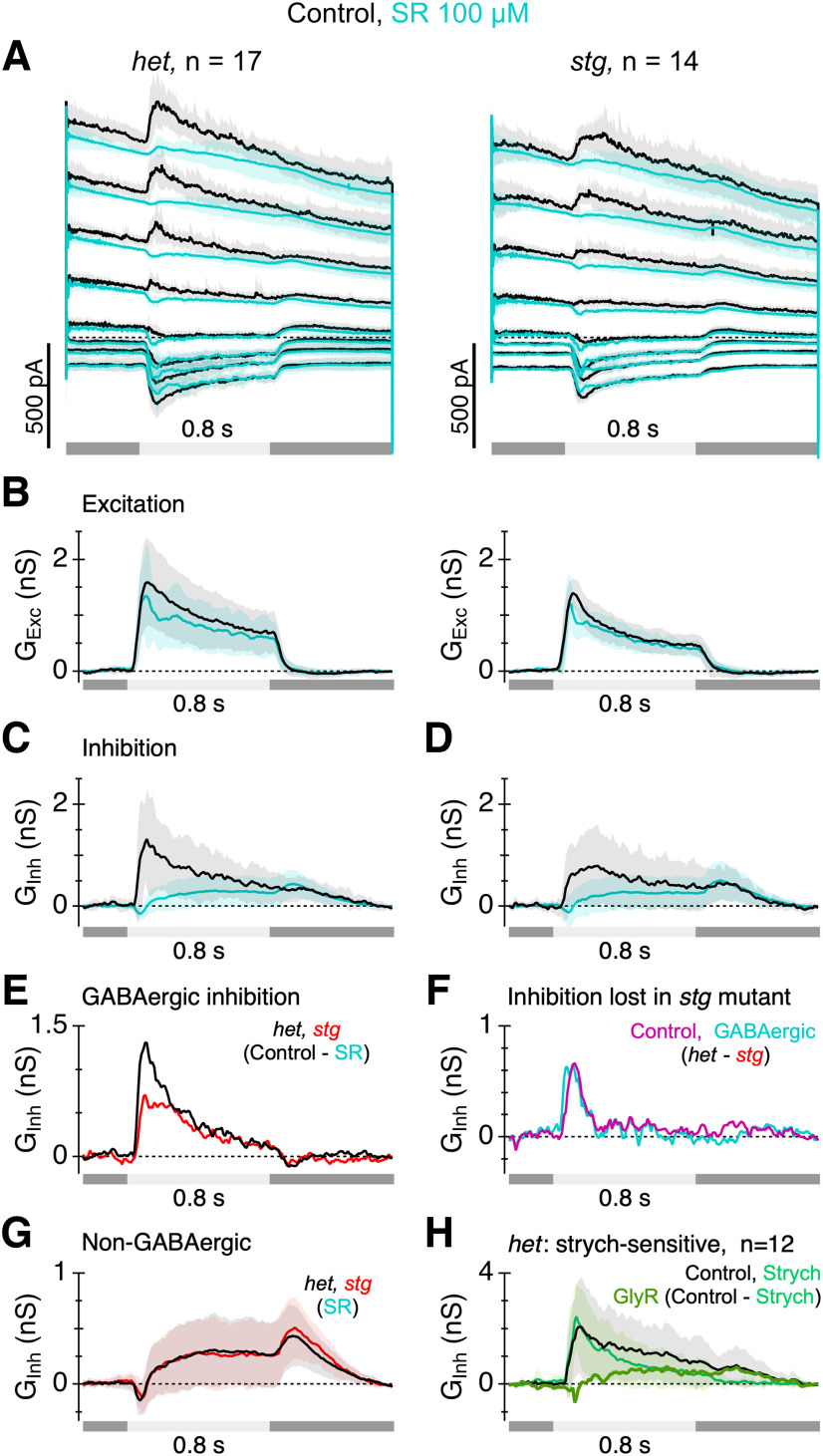
Loss of TARPγ2 produces circuit-specific reductions in inhibition. ***A***, Total membrane current during depolarizing voltage steps to holding potentials between −90 and +60 mV recorded in control (black) and in the presence of SR95531 (SR; 100 μM, cyan). Timing of the 175 μm diameter positive contrast stimulus spot is shown beneath the traces. Shading and error bars show ±1 SD. ***B***, Excitatory conductance calculated for the data in ***A***. ***C***, ***D***, Inhibitory conductance calculated for the data in ***A***. Left panels in ***A–D*** are from het SACs, right panels from *stg* SACs. ***E***, Net GABA_A_R-mediated conductance (control−SR) in *het* (black) and *stg* (red) SACs. ***F***, Comparison of the inhibitory conductance missing in *stg* SACs (Fig. 5*F*, *het* - *stg*, magenta) with the GABA_A_R-mediated conductance missing in *stg* SACs (Fig. 6*E*, *het* - *stg*, cyan). ***G***, The traces are replotted from ***C***, ***D*** (cyan traces) to compare the non-GABAergic (SR-resistant) inhibition in the *het* and *stg* amacrine cells. ***H***, Inhibitory conductance averaged from 12 ON-SACs in *het* retinas in control (black) and after addition of 1 μM strychnine (strych, green). The thick green trace shows the net conductance blocked by strychnine (control minus strychnine). Error bars and shading are ±1 s.d.

SACs receive inhibitory inputs from neighboring SACs (Lee and Zhou, 2006: Briggman et al., 2011; Ding et al., 2016) and other ACs (Millar and Morgan, 1987; Münch and Werblin, 2006; Briggman et al., 2011). Given the reduced excitatory input to SACs, we tested whether GABAergic input to the SACs was affected in the *stg* mutant by applying the GABA_A_ receptor (GABA_A_R) antagonist, SR 95531 (100 μM, SR; Fig. 6*A*). SR had little effect on excitatory input to SACs in the *het* or *stg* animals (Fig. 6*B*); however, it partially suppressed inhibitory inputs (Fig. 6*C*,*D*, amplitude 130 ms after flash onset significantly suppressed, paired *t* test, *het p* = 7.7 × 10^−6^, *n* = 17 cells, *stg p* = 3.9 × 10^−4^, *n* = 14 cells). The net GABA_A_R mediated conductance is shown in Figure 6*E* and was calculated by subtracting the inhibition in the presence of SR from the control before application of SR. Relative to control, the GABA_A_R conductance in *stg* SACs lacked a transient component at the beginning of the response (Fig. 6*E*), reminiscent of the difference in the unidentified inhibition observed in *het* and *stg* cells shown in Figure 5*F*. Therefore, we sought to compare the inhibition lost in the *stg* animals (magenta; Fig. 5*F*), with the GABA_A_R inhibition lost in the *stg* animals (Fig. 6*E*). To do this, we subtracted the GABA_A_R conductance in the *stg* animals from the GABA_A_R conductance in the *hets* (black trace minus red trace in Figure 6*E* shown as the cyan trace in Fig. 6*F*). For comparison, we have replotted the unidentified inhibition lost in the *stg* animals (magenta; Fig. 5*F*), as the magenta trace in Figure 6*F*. The remarkable quantitative agreement in the amplitude and time course of the traces in Figure 6*F* suggests that the inhibition that is lost in the *stg* SACs is most likely GABAergic. Note that there is no overlap in the cell samples contributing to the data in Figures 5 and 6.

The ON-SACs also received a substantial SR-resistant inhibitory input (Fig. 6*G*, cyan traces, replotted from Fig. 6*C*,*D* for comparison). The mean amplitude and time course of this SR-resistant inhibition were essentially identical in the *het* control and *stg* groups. It is noteworthy that the OFF-inhibition, seen at the termination of the light flash, was unaffected by SR (Fig. 6*C*,*D*) and was identical in the *het* and *stg* animals (Fig. 6*G*), indicating that the inhibitory OFF-inputs to ON-SACs were unaffected in the *stg* mutant. Given the presence of a SR-resistant inhibitory input to the ON-SACs, we tested whether inhibitory inputs to ON-SACs included a glycinergic component (Jain et al., 2022) by applying the glycine receptor antagonist 1 μM strychnine to a sample of 12 *het* ON-SACs (Fig. 6*H*). Strychnine had no effect on the initial peak of the inhibitory conductance but suppressed the conductance at later times (compare black and green traces, Fig. 6*H*, paired *t* test, *p* = 4.2 × 10^−3^, *n* = 12). The strychnine-sensitive conductance (control, strychnine; Fig. 6*H*, thick trace), had a similar amplitude and time course as the SR-resistant component (Fig. 6*H*). Note that the OFF-inhibition was completely suppressed by strychnine (Fig. 6*H*, *p* = 2.3 × 10^−2^). Overall, the results in Figures 5 and 6 demonstrate that the loss of TARPɣ2 has selective effects on retinal circuits. The ON inhibitory inputs to ON-SACs are partially suppressed, while OFF-inputs to ON-SACs, presumably mediated by a narrow-field glycinergic amacrine cell (Jain et al., 2022), are largely unaffected.

**Figure 7.**
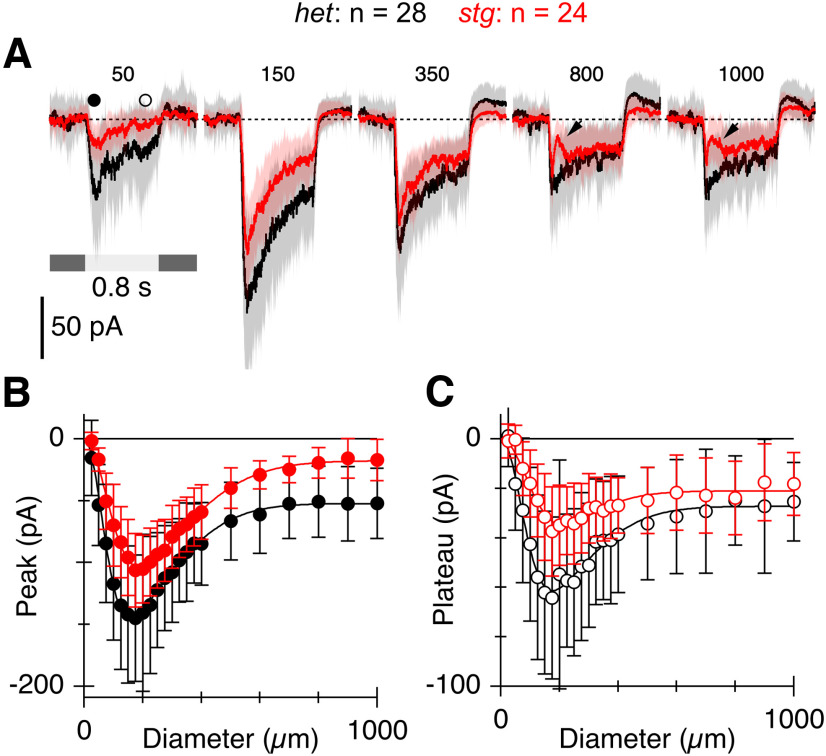
Absence of TARPγ2 does not affect ON-SAC receptive field size. ***A***, Average light-evoked EPSCs in ON-SACs from *het* (*n* = 28 cells) and *stg* retinas (*n* = 24 cells). EPSCs were elicited by centered light spots (diameters (μm) are shown above traces). ***B***, ***C***, Amplitudes of EPSCs versus stimulus diameter for the time points shown by the corresponding symbols in ***A***. Smooth lines show fits to a difference-of-Gaussians function.Error bars and shading are ±1 s.d.

The results thus far show that loss of TARPɣ2 has effects on both the excitatory and inhibitory inputs to ON-SACs. We recorded area-response functions to determine whether the absence of TARPɣ2 also affected spatial tuning of ON-SACs. The static area-response function for the SACs could be described by a concentric difference-of-Gaussians model (Fig. 7*A–C*, fitted lines; see Materials and Methods). Similar to the results above, the peak light-evoked EPSC for a spot diameter of 175 μm was reduced by ∼28% in the mutant SACs (*het* = −128 ± 49 pA, *stg* = −92 ± 33 pA, *p* = 0.0046; Fig. 7*B*). The extent of the center and surround receptive field components, as estimated from the widths of the fitted Gaussian functions, were similar in *het* and *stg* SACs, however, the strength of the surrounds differed. In *het* retinas, the surround suppressed the peak EPSC by 57 ± 20% (*n* = 28), compared with 75 ± 13% (*n* = 24, *p* = 3.7 × 10^−4^) suppression in the *stg* SACs. Interestingly, surround inhibition produced a transient suppression of the EPSCs at the onset of the light flash in the *stg* mutant that was not evident in *het* retinas (Fig. 7*A*, arrows). These effects hint at subtle changes presynaptic to the SAC, but were not examined further. Overall, the data indicate a decrease in the excitatory drive to SACs and subtle, circuit-specific effects on the inhibitory inputs.

**Figure 8.**
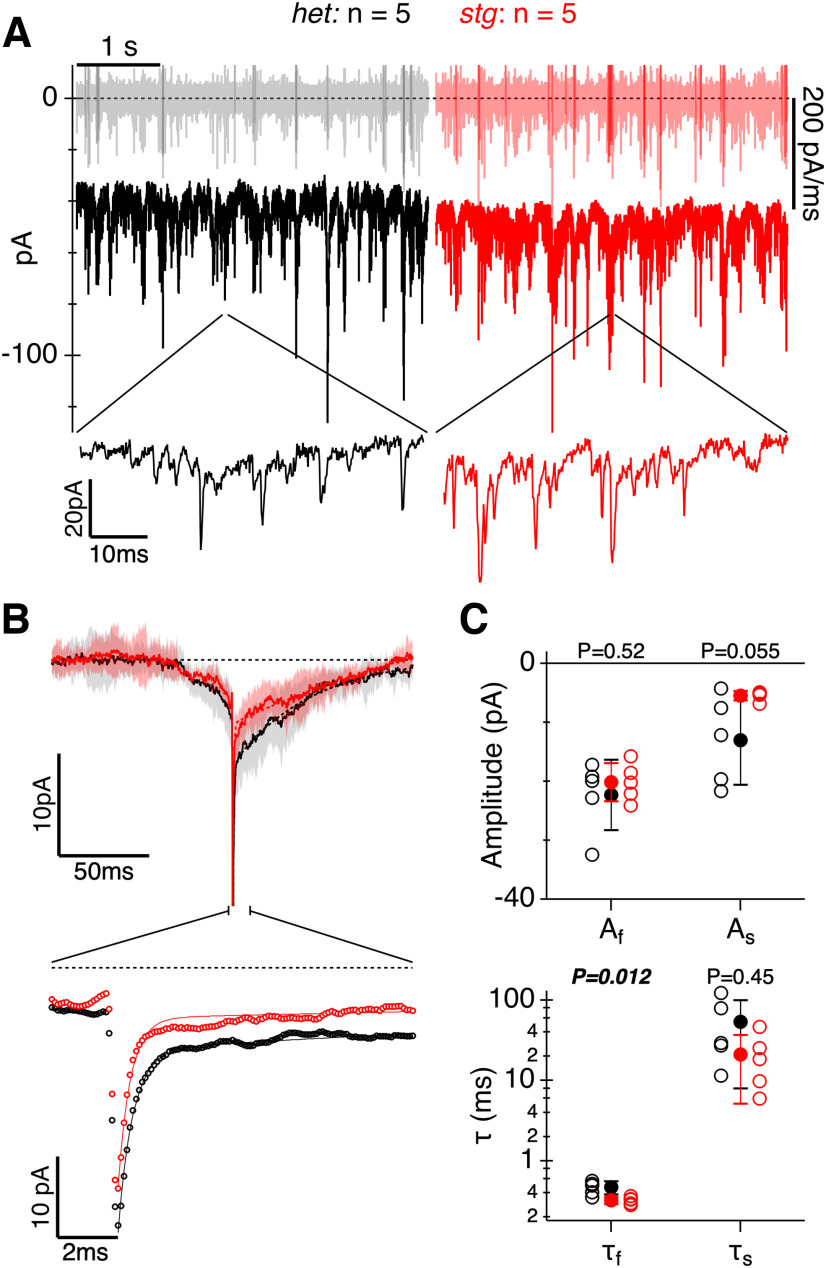
Spontaneous EPSCs (sEPSCs) are altered in the absence of TARPγ2. ***A***, Sample current records from a het (black) and *stg* retina (red). Lighter traces at 0 pA show the derivative of the current records that were used to threshold and detect spontaneous events. Lower traces show detail for sample segments on an expanded time-base. ***B***, Average sEPSCs generated from 580 events in five het SACs and 638 events in five *stg* SACs. The detection threshold was 5 SD (see Materials and Methods). Lower records show double exponential fits to the decay of the average sEPSCs. ***C***, The amplitude of the fast component of the sEPSCs was unchanged. The amplitudes of the slow component for the *stg* SACs was much less variable than in het SACs, but the difference in means was not statistically significant. The fast decay component was significantly faster in *stg* SACs, but the slow decay time-constant was unchanged. *p*-values for an unpaired *t* test are shown above the parameters in the two panels (see also Extended Data Table 2-1). Error bars and shading are ±1 s.d.

**Figure 9.**
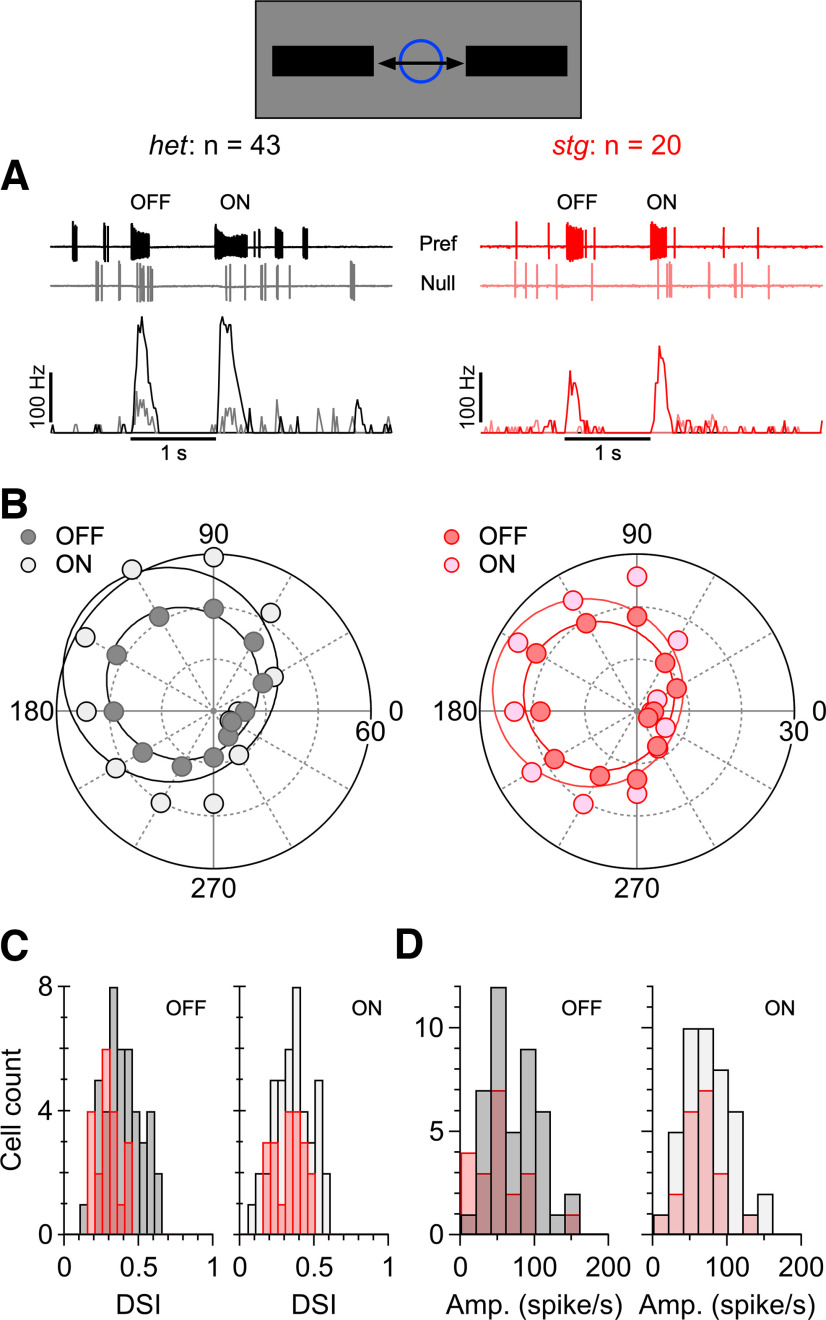
Direction selectivity is largely unaffected by the absence of TARPγ2. Extracellular spikes were elicited by a dark bar moving in 12 directions through the receptive fields of ON-OFF direction-selective ganglion cells (DSGCs). The stimulus bar was 1 mm long, 200 μm wide and moved at 1 mm/s. The approximate dimension of the DSGC receptive field (blue circle) relative to the stimulus bar is shown in the schematic. ***A***, Sample extracellular spike recordings from ON-OFF DSGCs in a het (black) and *stg* mutant retina (red) for preferred and null direction stimuli. Lower panels show peristimulus spike-time histograms (PSTHs) accumulated from 40 trials in each cell. ***B***, Directional tuning for the OFF (leading edge) and ON (trailing edge) responses shown in ***A***. Distance from the origin represents the peak of the respective PSTHs. Solid lines show fits to the von Mises function (see Materials and Methods). ***C***, Distributions of the direction-selectivity index (DSI), here defined as the normalized length of the vector sum of the tuning function illustrated in ***B***. ***D***, Amplitude distributions for the tuning functions calculated from the von Mises fits to the data in ***B***.

**Figure 10.**
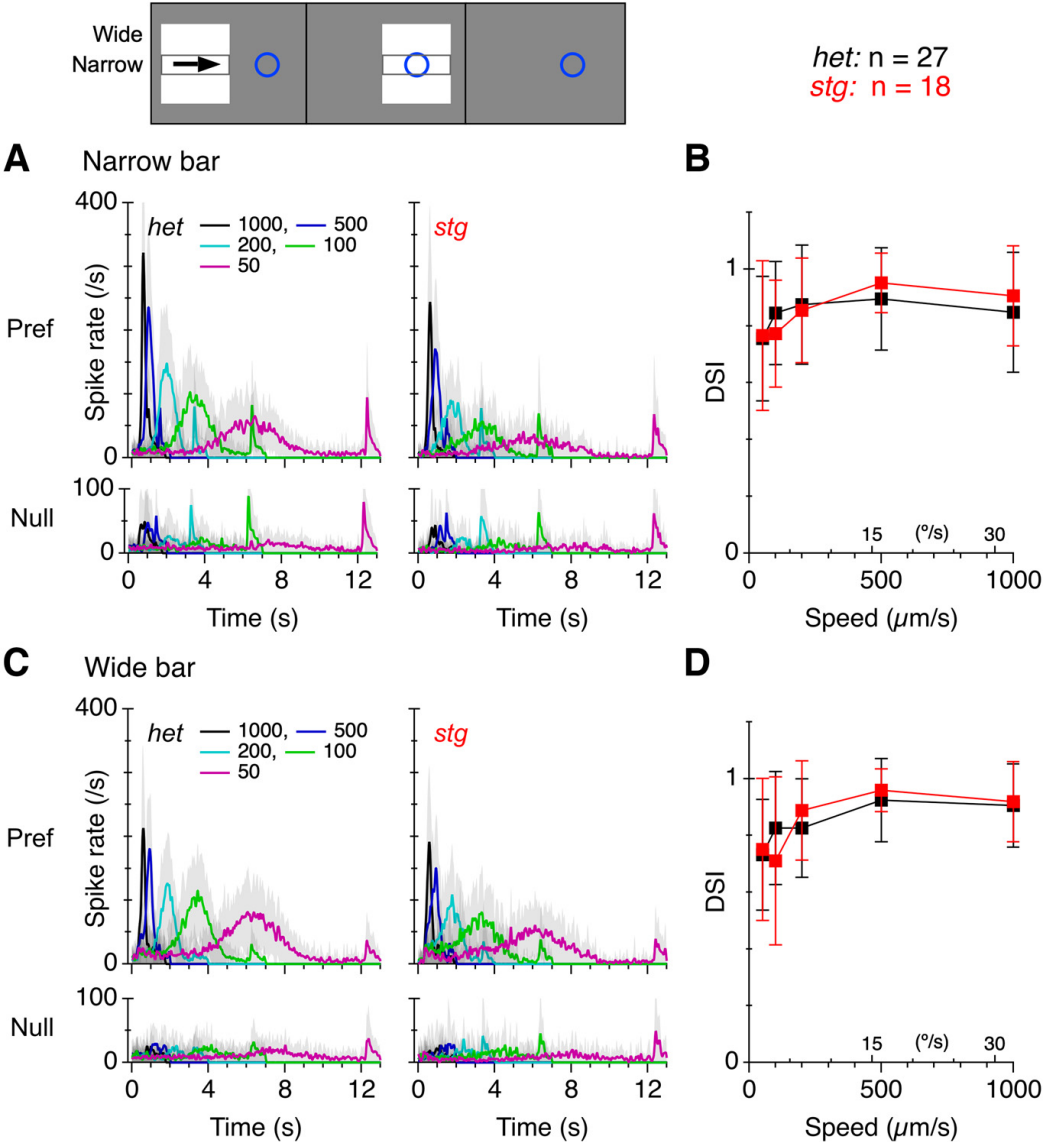
Directional tuning as a function of speed is unaffected by the absence of TARPγ2. PSTHs calculated for spikes elicited by narrow and wide bright bars moving through the receptive fields of ON-OFF direction-selective ganglion cells (DSGCs) in the preferred and null directions. The approximate dimension of the DSGC receptive field (blue circle) relative to the stimulus bars is shown in the schematic. The bars move, stop, and then disappear. ***A***, PSTHs of extracellular spike recordings from ON-OFF DSGCs elicited by a narrow bar moving at a range of stimulus speeds (μm/s, shown in the legend) for both preferred (pref) and null (null) directions. A transient OFF-response is seen when the bar disappears at the end of the motion. ***B***, DSIs, (Pref-Null)/(Pref+Null), as a function of stimulus speed for the data in ***A***. ***C***, ***D***, Same format as ***A***, ***B*** but for a wide-bar stimulus. Error bars and error shading indicate ±1 s.d.

**Figure 11.**
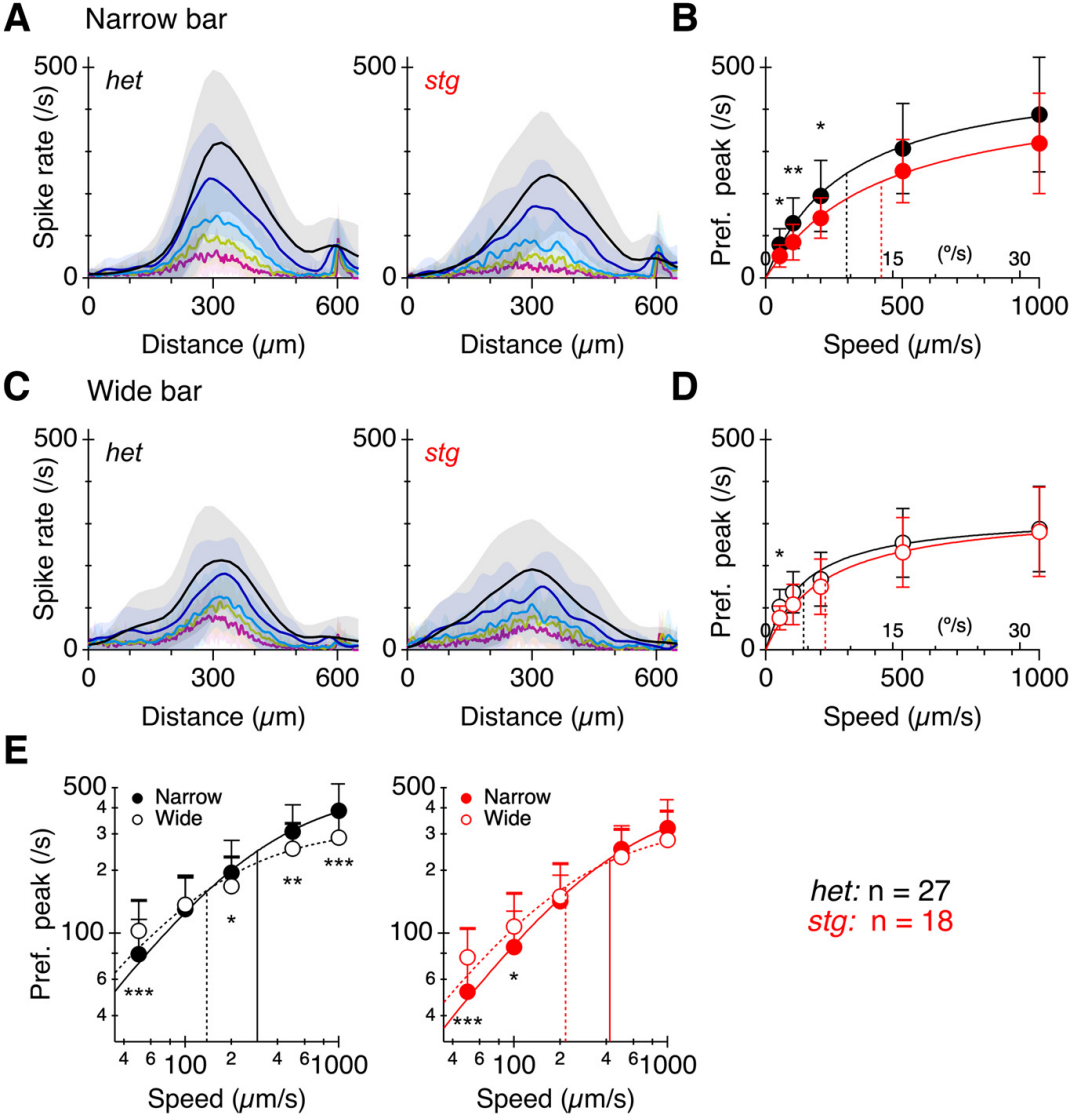
Spatial tuning is reduced in the absence of TARPγ2. Spike-responses from DSGCs. Error bars and trace shading show SDs. ***A***, PSTHs from Figure 10*A*, narrow bar, plotted against stimulus position. ***B***, Peak spike-rate as a function of stimulus speed for the data in ***A***. The smooth lines show fits to the Hill equation with *n* fixed at 1 (see Materials and Methods). The vertical lines show the half-maximal speeds from the fits. ***C***, ***D***, Same format as ***A***, ***B*** but for the wide-bar stimulus. ***E***, Narrow bar and wide bar data re-plotted from ***B***, ***D*** on log-log coordinates. *p < 0.05, **p < 0.01, ***p < 0.001 with Sidak’s multiple comparisons test (see also Extended Data Table 2-1).

A distinguishing feature of the excitatory input to SACs is the presence of continuous spontaneous EPSCs (sEPSCs) under background illumination (Taylor and Wässle, 1995; Petit-Jacques et al., 2005; Petit-Jacques and Bloomfield, 2008; Stincic et al., 2016; Stincic et al., 2018). These excitatory inputs are mediated primarily by AMPARs (Zhou and Fain, 1995; Firth et al., 2003; Petit-Jacques and Bloomfield, 2008). The continuous input arises from ON-bipolar cells and can be suppressed by decreasing illumination, for example, at the end of a light flash (Taylor and Wässle, 1995; Petit-Jacques et al., 2005; Petit-Jacques and Bloomfield, 2008; Stincic et al., 2016; Stincic et al., 2018). We tested whether the reduction in the light-evoked EPSCs might be because of a lower postsynaptic response by comparing the amplitude of spontaneous EPSCs in SACs from *stg* and *het* control mice. Spontaneous EPSCs (sEPSCs) were detected by differentiating the current record (Fig. 8*A*, upper traces) and thresholding for the most rapid events (see Materials and Methods). Individual events can be discerned in the raw records (Fig. 8*A*, lower traces). The frequency of the events was the same in *het* (14.8/s) and *stg* (13.8/s) mice. Larger, rapid sEPSCs appear to be superimposed on slower fluctuations in the membrane current. Average sEPSCs showed a complex time course with an initial slow increase in the inward current preceding the rapid events detected by the threshold (Fig. 8*B*, top panel). The decay-rate of the average sEPSCs was measured by fitting a double exponential to the decay phase (Fig. 8*B*, lower panel). The rate of the initial rapid decay was ∼35% faster in cells from the *stg* mutants compared with *hets* (Fig. 8*C*; Tau_f_ = 464 ± 72 μs *het*, 305 ± 75 μs *stg*, *n* = 5 cells, *p* = 0.012), whereas the slow time-constant of the sEPSC decay was unchanged (Tau_s_ = 53.0 ± 41.2 ms *het*, 33.0 ± 29.8 ms *stg*, *n* = 5 cells, *p* = 0.45). The amplitude of the fast decay component of the sEPSCs was unchanged (*p* = 0.523), while the slightly reduced amplitude of the slow component was not statistically significant (*p* = 0.086; Fig. 8*C*). The results indicate that the reduced amplitude of light-evoked EPSCs in *stg* SACs shown above cannot be explained simply by a reduced postsynaptic conductance, although these experiments were underpowered to detect a small effect size.

### Absence of TARPɣ2 has modest effects on directional tuning of direction-selective ganglion cells

SACs provide the asymmetric GABAergic input to direction-selective ganglion cells (DSGCs) that is critical for their directional responses. We sought to determine whether the reduced excitatory input to the SACs resulting from the loss of TARPɣ2 might affect the strength of directional signals in the DSGCs. To this end, we identified ON-OFF DSGCs by recording extracellular action potentials and measured responses to bars drifting across the receptive field in different directions. We calculated the peristimulus spike-time histograms (PSTHs) from three to four stimulus trials in each direction (Fig. 9*A*). The spike-rates, measured from the PSTHs at a fixed time point near the peak during preferred direction stimuli, were used to calculate directional tuning functions for the OFF (leading edge) and ON (trailing edge) components of the responses (Fig. 9*B*). The strength of the directional response was quantified as the direction-selectivity index (DSI), which was measured as the normalized length of the vector sum of the responses in all 12 directions. As defined, the DSI varies between 0 and 1 (weakest to strongest).

Directional responses in DSGCs persisted in the TARPɣ2 mutant (Fig. 9*A*,*B*, red); however, examination of the tuning indicated subtle effects (Fig. 9*C*,*D*). The average DSI for the OFF-response was lower in the *stg* mutant retinas than in *het* retinas (*het*: 0.38 ± 0.12, *n* = 43; *stg*: 0.29 ± 0.08, *n* = 20, *p* = 1.81 × 10^−3^), but the corresponding ON-responses were unchanged (*het*: 0.35 ± 0.13, *stg*: 0.33 ± 0.10, *p* = 0.385). Similarly, the amplitude of the OFF-responses tended to be smaller in *stg* DSGCs (*het*: 72 ± 33 Hz, *stg*: 52 ± 33 Hz, *p* = 0.028), while the ON responses were unchanged (*het*: 74 ± 32 Hz, *stg*: 63 ± 25 Hz, *p* = 0.192). Finally, the alignment of the preferred-null axes for the ON and OFF components was less precise in the *stg* mutant retinas. In DSGCs from *het* retinas the ON and OFF preferred directions differed by 7.8 ± 6.8° (*n* = 43), whereas the misalignment in the *stg* mutant retinas was almost 2-fold larger at 14.6 ± 10.4° (*n* = 20, *p* = 5.26 × 10^−3^). Overall, these results indicate relatively minor changes in ON-OFF DSGC tuning in *stg* mice.

### Directional, spatial and speed tuning of DSGCs is preserved in *stg* mutants

ON-OFF DSGCs display selectivity for small objects by virtue of surround inhibition mediated by SACs as well as other AC types (Hoggarth et al., 2015). Since SACs in *stg* retinas showed a reduced excitatory input, we tested whether the absence of *TARPɣ2* leads to changes in the directional or spatial tuning of the DSGCs. Narrow (600 × 100 μm) and wide (600 × 600 μm) bright bars were drifted across the receptive field of DSGCs in the preferred and null directions at a range of speeds (Fig. 10*A*,*C*). The direction-selectivity index (DSI; see Materials and Methods) was similar in *stg* and *het* control retinas and was invariant to changes in speed or bar width (Fig. 10*B*,*D*).

We examined how the loss of TARPɣ2 might affect spatial tuning and speed tuning (Fig. 11). For narrow bars, responses in *stg* DSGCs were smaller than in *het* controls. Overall, there was a significant effect of genotype on peak spike rate (RM two-way ANOVA, genotype, *p *=* *0.0086; speed *p* ≤ 0.0001, interaction *p* = 0.7433) and *post hoc* comparisons showed significant differences between *stg* and *het* DSGCs for the three lowest speeds tested (Fig. 11*B*). In contrast, no differences were observed between *stg* and *het* DSGCs for wide bar stimuli (two-way ANOVA, genotype *p *=* *0.1685; speed *p* ≤ 0.0001, interaction *p* = 0.9031; Fig. 11*D*). These results indicate an overall reduction in the excitatory drive to DSGCs in the *stg* mutant.

Spatial tuning of DSGCs was altered in the *stg* mutants. Whereas in *het* control retinas, responses to wide bars were suppressed relative to narrow bars at higher speeds, such surround-suppression was not evident in the *stg* retinas (Fig. 11*E*). For the *het* cells, simple main effects analysis showed no overall effect of bar width on spike rate (two-way ANOVA, bar width, *p *=* *0.0526; speed *p* < 0.0001) but there was a significant interaction between bar width and speed (*p *<* *0.0001). *Post hoc* comparisons showed that bar width suppressed peak spike rate for the highest speed but not at low speeds, indicating speed-dependent surround suppression. In the *stg* mutant, bar width had no significant effect on spike rate (two-way ANOVA, bar width, *p *=* *0.9402; speed *p *<* *0.0001) but there was a significant interaction between bar width and speed (*p *<* *0.03). *Post hoc* analysis showed wider bars did not suppress responses at higher speeds in *stg* retinas. Together, these results indicate that surround suppression in DSGCs is velocity dependent, and suggest that surround suppression is weaker in the *stg* mutants.

Speed tuning was quantified by plotting the peak response during preferred direction stimulation versus stimulus speed (Fig. 11*B*,*D*). In both *het* and *stg* retinas, half-maximal responses were reached at ∼2-fold higher speeds for narrow bars versus wide bars, indicative of broader dynamic range but lower threshold sensitivity for narrow bars (Fig. 11*E*). Overall, the data indicate that speed tuning is largely preserved in DSGCs in the *stg* mutants.

## Discussion

AMPARs are expressed at most excitatory synapses in the retina, yet little is known about which auxiliary subunits are associated with which AMPAR subunits. Here, we show that TARPɣ2 is expressed at outer and inner retinal synapses. Quantitative results in mice indicate highest expression at the level of stratification of the OFF-SACs and ON-SACs. The relative enrichment of TARPɣ2 protein in SACs is mirrored at the transcript level for mouse and human retina. Expression of GluA2, GluA3 and GluA4 was significantly reduced in the inner retina of mice lacking TARPɣ2, whereas GluA1 expression was unchanged. Consistent with our immunohistochemical data, functional studies revealed reduced excitatory currents and altered mEPSCs in ON-SACs. However, direction-selectivity was only modestly affected for the stimulus conditions tested, suggesting that the circuit mechanisms that mediate direction selectivity are robust to modest changes in AMPAR expression.

### TARPγ2 is required for normal GluA expression levels

We found evidence for reduced expression of GluA2, GluA3 and GluA4 in the IPL of *stargazer* mice whereas there was no effect on GluA1 expression. Early studies in this mouse line showed near complete loss of AMPARs in cerebellar granule cells (Chen et al., 2000). However, in most other neurons, knock-out of a single TARP only partially reduced AMPAR levels (Rouach et al., 2005; Menuz and Nicoll, 2008; Menuz et al., 2008; Barad et al., 2012; Shevtsova and Leitch, 2012; Adotevi and Leitch, 2017), similar to our findings. These modest effects of TARPɣ2 knock-out have been explained by functional redundancy. For example, in cerebellar Golgi cells, single knock-out of -ɣ2 or -ɣ3 had no effect on AMPAR currents, whereas currents were abolished in a -ɣ2/3 double mutant (Menuz et al., 2008). Such functional redundancy prevents the severe phenotypes and early postnatal lethality associated with the double knock-outs (Menuz et al., 2008). The presence of residual GluA protein throughout the inner retina and residual AMPAR currents in ON-SACs and ON-OFF DSGCs suggests that other TARPs, or AMPAR auxiliary proteins such as CKAMPs or cornichons, may be present (Hansen et al., 2021). We did not detect TARPɣ4 or TARPɣ8 in the *stg* mouse, but single-cell transcriptomic data points to TARPɣ3, TARPɣ5, or TARPɣ7 as possible candidates for such compensation (Yan et al., 2020a, GEO GSE149715, data not shown). An alternate possibility is that a proportion of retinal AMPARs are TARPless as has been shown in other brain regions (Bats et al., 2012).

### AMPAR distribution in mouse inner retina

Prior studies have shown that GluA1–GluA4 are expressed in the mouse IPL (Haverkamp et al., 2000). Our results provide a quantitative assessment of how AMPAR subunit expression varies with IPL depth. We found that the levels of GluA1 and GluA2 are relatively low in s5 of the IPL (80–100% depth; Fig. 4), the region where rod bipolar cells stratify. In accordance with this finding, the major postsynaptic partners of the rod bipolar cells, the AII ACs and A17 ACs, are known to express calcium-permeable AMPARs, which typically lack the GluA2 subunit (Chávez et al., 2006; Osswald et al., 2007; Diamond, 2011). GluA1 and GluA2 are also expressed at low levels in S5 of the macaque IPL (Ghosh et al., 2001) suggesting that the basic patterns of AMPAR expression may be conserved across species, at least for neurons associated with the rod pathway.

### AMPAR composition and function in starburst amacrine cells

The reduction in AMPAR expression in the *stargazer* mutant was nonuniform across IPL depth, with the largest reductions seen at the level of the SAC dendrites. These data align with findings at the transcript level showing enrichment of *Cacng2* in SACs relative to other amacrine cell types in both mouse and human retina. The reduction in light-evoked EPSC amplitude in *stg* SACs further supports a role for TARPɣ2 in normal synaptic AMPAR expression in these cells. What is the expected functional impact of TARPɣ2 on excitatory inputs to SACs? In addition to its involvement in increasing synaptic trafficking and localization, TARPɣ2 can decrease the rate of channel deactivation and desensitization, increase rate of recovery from desensitization, and increase single channel conductance and glutamate affinity (Yamazaki et al., 2004; Priel et al., 2005; Turetsky et al., 2005; Jacobi and von Engelhardt, 2021). Indeed, the rapid phase of the sEPSC decay was significantly faster in *stg* SACs compared with het controls (Fig. 8), consistent with the idea that TARPɣ2 has a role in slowing the rate of channel deactivation. Moreover, the average amplitude of the slow phase of the spontaneous EPSCs was smaller in SACs from *stg* mice (Fig. 8*C*), and although this effect did not reach significance, it is consistent with the immunohistochemical evidence for reduced AMPAR expression in the *stg* mouse.

Excitatory inputs to mouse SACs are mediated primarily by AMPARs (Firth et al., 2003; Petit-Jacques and Bloomfield, 2008). However, there are apparently two pharmacologically distinct excitatory current components in rabbit ON-SACs: a noisy, sustained component that is sensitive to low concentrations of NBQX (750 nm), and a more transient component that persists in the same concentration of NBQX (Oesch and Taylor, 2010). One interpretation of this finding is that SACs express different populations of AMPARs with distinct pharmacology because of the presence or absence of TARPs. Indeed, NBQX has been shown to be less effective at blocking AMPARs that are in complex with TARPɣ2 compared to those without it (MacLean et al., 2014; Devi et al., 2016). Our analysis of ON-SAC sEPSCs lend further support for AMPAR heterogeneity in ON-SACs. Average sEPSCs exhibited complex kinetics, with a slow rise preceding a rapid event, followed by a dual exponential decay. Events were detected by thresholding the differential of the current signal (dI/dt), which will favor fast events, and will tend to select for larger events. Two lines of evidence suggest the presence of a heterogeneous population of postsynaptic receptors. (1) The initial slow rise in current preceding the fast sEPSC is inconsistent with synchronous activation of a single population of receptors exposed to the same rapid vesicular transmitter release. A possible explanation is that fast sEPSCs are temporally correlated with other release processes that produce slower sEPSCs. (2) The time constant of the fast decay component was faster in the *stg* mutant whereas the slow decay component was unchanged. This result is inconsistent with the EPSCs arising from a homogeneous population of receptors that are affected in the mutant. Altogether, the current results and previous pharmacological findings point to two EPSC components in SACs that arise from AMPARs with distinct kinetic properties that are temporally correlated by presynaptic release. The functional contributions of these two AMPAR components remains to be determined.

### AMPAR composition and function in DSGCs

Excitatory input to ON-SACs was reduced and it is possible that excitatory inputs to DSGCs were also affected by the loss of TARPɣ2. Reduced spiking is evident for the OFF-inputs but interpretation of such effects is confounded by potential effects on inhibitory circuits. Despite the reduced excitatory input to ON-SACs in stargazer mice, directional responses were preserved in ON-OFF DSGCs. Consistent with this finding, persistence of direction-selectivity has been shown in a mouse model where SACs have reduced and less asymmetric GABA release between preferred and null direction stimulation (Pei et al., 2015). Furthermore, inhibitory outputs from SACs are saturated at relatively low contrasts and thus the high contrast stimuli used here presumably produced sufficient GABA release to maintain normal DS responses (Lipin et al., 2015). In this light, further studies may be needed to determine whether the effects of TARPɣ2 on direction-selectivity vary with stimulus contrast. Interestingly, the alignment of the preferred directions for the ON and OFF responses was less precise in stargazer mice compared with *het* controls suggesting some mild perturbation of circuit wiring in the stargazer mutant mouse during development.

The absence of TARPɣ2 had specific effects on select retinal circuits. The loss of TARPɣ2 resulted in the loss of a transient GABAergic input to ON-SACs at the onset of a light-flash, leaving more sustained GABAergic and glycinergic inputs unaffected (Fig. 6*E–H*). Similarly, glycinergic inhibition at the termination of a light flash was unaffected. The glycinergic inputs appeared to be similar to those described previously (Jain et al., 2022) and are thought to arise from narrow-field amacrine cells. Thus, our results indicate a selective effect on the ON-pathway-driven input to ON-SACs. Finally, spatial tuning in *stg* mutants was affected. The results align with previous work showing that DSGCs are tuned to respond more strongly to smaller objects because of surround inhibition (Hoggarth et al., 2015); however, a novel finding is that such spatial selectivity is velocity-dependent because at low speeds the wide bar produced larger responses than the narrow bar, consistent with a weaker surround. This result might be explained if the surround inhibition were relatively transient compared with excitation. Conversely, at higher speeds the wide bar suppressed responses more strongly than the narrow bar, but most importantly for the present purposes, this surround-suppression was not seen in the *stg* mutants (Fig. 11*E*). Overall, the loss of TARPɣ2 produced subtle functional effects that appeared to be confined to specific retinal circuits, presumably reflecting selective expression within retinal neurons. Our analysis necessarily focuses on the ON-pathway, as the ON-SACs are displaced to the ganglion cell layer and thus are accessible for recording. Although OFF inputs remain directional in the absence of TARPɣ2, it remains to be seen whether there are other effects on directional-responses in the OFF-pathway. Similarly, TARPɣ2 is not expressed exclusively in direction-selective circuits, thus further studies are needed to determine the impact of the loss of TARPɣ2 on other retinal neurons.

## Synthesis

Reviewing Editor: Matthew Grubb, King’s College London Decisions are customarily a result of the Reviewing Editor and the peer reviewers coming together and discussing their recommendations until a consensus is reached. When revisions are invited, a fact-based synthesis statement explaining their decision and outlining what is needed to prepare a revision will be listed below. The following reviewer(s) agreed to reveal their identity: Tomomi Ichinose.

Synthesis:

Both Reviewers view the manuscript positively, but have several issues they feel need to be addressed. Please ensure the article is extensively revised to address each of the Reviewers’ comments as outlined below. After consultation, we would like to clarify the following two issues:

1) Reviewer 1 asks for additional OFF SAC recordings to support the conclusion that the OFF component of IPSCs was not affected by the mutant. We agree that these recordings would be a valuable addition to the manuscript; however, if they are not feasible the authors should tone down their claims regarding this OFF pathway (see also Reviewer 2’s comments on OFF responses).

2) The immunohistochemical data in Figure 4 require additional experiments and/or analysis to convincingly demonstrate a reduction of GluA2/3/4 in the IPL of the TARPγ2 KO. Authors should either add additional quantitative data, and/or should present additional analyses using a convincing reference staining region in order to calibrate and compare the relative labelling intensity across samples.

Reviewer 1:

The authors investigated how the transmembrane AMPA receptor

regulatory proteins (TARPs) affect visual signaling in the retina. TARPs are critical for the AMPA receptor (AMPAR) expression and signaling. Among six TARPs, the authors selected TARPγ2 (Stargazin) and investigated the expression and functions. Immunohistochemistry revealed that TARPγ2 is expressed in the inner plexiform layer (IPL) and colocalized with CHAT bands. The authors targeted starburst amacrine cells (SACs) and direction-selective ganglion cells (DSGCs) to examine physiological functions in the presence and absence of TARPγ2s. In SACs, both excitatory and inhibitory postsynaptic currents (EPSCs and IPSCs) were reduced in TARPγ2-knockout mice. However, the spatial tuning of SACs, spontaneous EPSCs in SACs, and direction-selectivity in DSGCs were only slightly affected in TARPγ2-knockout mice, suggesting a little effect of TARPγ2 on retinal signaling.

Figures are well-presented, and descriptions are straightforward. Physiological results show that TARPγ2-knockout minimally affected the direction-selective signaling, which is consistent with other references. Since TARPs have not been investigated in the retina, the article shows the TARPγ2 data for the first time, which may be crucial in the field. However, there are some concerns that need clarification:

1. The authors claim that TARPγ2-knockout reduced the GluA2, GluA3, and GluA4 in the IPL. However, immunohistochemistry is not quantitative, and the results in Figure 4 are not convincing. Did TARPγ2-knockout reduce the AMPA receptor surface expression? Or did it change receptor assembly? Mechanisms and results are not convincing regarding the AMPA receptor in the TARPγ2-knockout mice.

2. In Figure 5, both EPSCs and IPSCs were reduced in TARPγ2-knockout mice. The kinetic differences are clearly shown in the reduced IPSCs. However, the reduced EPSCs exhibited similar kinetics to the control WT mice. Were there any kinetic differences between the control and mutant? Each subunit might show different kinetics; this analysis might indicate reduced kinetics differences.

3. The logic underlying Figure 6E-F is confusing. Did the authors conclude that the reduced IPSCs by TARPγ2-knockout are through GABA-A receptors? It should be partly true, but they may also be through other receptors, such as glycinergic. If IPSCs (control - TARPγ2-knockout) are exclusively GABA-A mediated, SR should not change the IPSCs_TARPγ2-knockout. However, SR still reduced the IPSCs_ TARPγ2-knockout, the IPSCs (control - TARPγ2-knockout) might include other components.

4. The authors claim that the off component of IPSCs was not affected by the mutant. However, it requires more data to conclude this. At least, recording from OFF SACs to examine the off signaling pathway. However, OFF bipolar cells may contain AMPA receptors, which may be involved in the TARPγ2-regulation.

5. The authors examined the DSGC PSTHs (spikes) in response to moving stimuli. Because TARPγ2 is expressed in the broad regions in the IPL, AMPA receptors in DSGCs might also be affected. Therefore, DSGC-PSTHs were affected both by SACs’ and DSGCs’ TARPγ2. This point should be described and discussed.

6. Although some AMPA-R subunits were reduced, overall signaling in SACs and DSGCs was hardly affected. The authors interpreted it as the overlapping effect of other TARPs. Did a compensatory effect potentially occur to rescue their signaling?

Minor

1. Page 13 Line 3: correct “moreso”.

2. The amplitude of sEPSCs was analyzed. How about the frequency of the sEPSCs in the mutant?

Reviewer 2:

In the manuscript entitled “TARPγ2 is required for normal AMPA receptor density and function in direction-selective circuits of the mammalian retina”, the authors investigate the distribution of TARPgamma2 immunoreactivity in the IPL of different mammalian species with a focus on mouse and AMPAR distribution when the TARP subunit is missing. Furthermore, electrophysiological features of starburst amacrine and direction selective ganglion cells were compared between wildtype and stargazer mice to gain insights into the functional role of TARPgamma2.

This work advances our understanding of AMPA receptor auxiliary subunits in the central nervous system and forms a robust basis for future analyses of TARP subunits in the retina. Furthermore, it adds valuable pieces to the puzzle of functional and structural components of one of the most prominent retinal circuitries to encode direction-selective responses.

The study was well performed and includes compelling data from challenging experiments. Also, the manuscript reads well. What follows is a collection of comments which addresses mostly minor points which may be considered by the authors to perhaps improve some aspects of the paper.

As suggested by the immunostainings, the expression patterns appear largely conserved across the four species included in the study, in a sense that TARPgamma2 appears to be associated with starburst amacrine cells. However, the authors may want to soften their generalizing statement “with highest expression” at the beginning of the Discussion section. The quantification has been performed in mouse only. And qualitatively, the macaque immunostaining shown in Fig. 1F strongly suggests that considerably high levels of TARP are found in another population of cells, at expression levels possibly comparable to starburst cells. From my perspective, the two strongly immunoreactive bands are too broad to exclusively originate from starburst dendrites. Either the tissue has not been properly aligned for sectioning (which does not seem to be the case based on the DIC image) or there is another set of cells to consider, like amacrine cells described in Majumdar et al https://doi.org/10.1002/cne.21666 or AC/GC types with similar stratification properties.

To clarify this issue, the authors could add an immunostaining of TARPgamma2 combined with ChAT in macaque sections. I don’t think that this data point is strictly necessary for the study, but it would allow more concise statements regarding the protein distribution relative to the ChAT bands and strengthen the study as a basis for potential follow-up research projects in primate retina.

Fig. 3, I recommend showing individual channels of the insets (together with the merged channels) so that the reader can better assess the colocalization pattern.

For Fig. 5F, the text reads “Off-driven inhibitory conductance at the end of the light flash was unaffected”. However, that component is not apparent in the stargazer trace of this panel. It is readily visible in Fig. 6D and generally more obvious during application of SR in wt (and stg to some extent), suggesting that it might depend on the relative strength of GABA-mediated inhibition in a given cell and condition. Either way, I recommend rephrasing that section and/or refer to Fig.6 in terms of OFF inhibition in stg.

In multiple figures, specifically obvious in Fig 6, the shaded sd areas have been cut off (or blocked by other figure parts in one instance). I suggest including the complete areas for proper display of the data.

The description of data in Fig. 6H would also benefit from a more explicit description regarding the animals and data display, currently using a rather confusing label combination of Ctrl, Control, and Con while panel H is part of the “stg column”, without that use of control/wildtype animals is specifically mentioned in the corresponding Results text section.

In the Methods section, please report the actual values for the “optimal z interval”

Fig 8C legend should read The fast decay component was significantly faster in stg SACs
