# Supplementary material for: TARPγ2 Is Required for Normal AMPA Receptor Expression and Function in Direction-Selective Circuits of the Mammalian Retina
Source: eNeuro. 2023 Aug 10;10(8):ENEURO.0158-23.2023. doi: 10.1523/ENEURO.0158-23.2023 (PMC10431237; doi:10.1523/ENEURO.0158-23.2023)
Supplement: Extended Data Table 1-1 — Summary of primary antibodies used in this study. Download Table 1, DOC file [file enu-eN-NWR-0158-23-s02.doc]

**Extended Data Table 1-1. Statistical summary table**

| **Figure** | **Data structure** | **Test type** | **n / N**  n= number of cells  N = number of mice | ***P* value** | **Power** |
| --- | --- | --- | --- | --- | --- |
| 2c | Normal | paired t-test | *N = 6* | *P*=0.0039 |  |
| 4 | Normal | unpaired t-test, Bonferroni correction for multiple comparison | *wt, N = 6*  *stg, N = 6* | *GluA1 OFF ChAT, P*=0.887  *GluA1 ON ChAT, P*=0.579  *GluA2 OFF ChAT, P*=0.003  *GluA2 ON ChAT, P*=0.006  *GluA3 OFF ChAT,P<*0.0001  *GluA3 ON ChAT, P=*0.001  *GluA4 OFF ChAT,P=*0.003  *GluA4 ON ChAT, P=*0.001  *PSD95 OFF ChAT, P=*0.730  *PSD95 ON ChAT, P=*0.831  *GABAAR OFF ChAT, P=*0.643  *GABAAR ON ChAT,P=*0.917 |  |
| 5d | Normal | unpaired t-test (Welch’s correction) | *het: n = 54, N = 22; stg: n=23, N = 10* | *P*=2.6x10^-6^ |  |
| 5f | Normal | unpaired t-test (Welch’s correction) | *het: n = 54, N = 22; stg: n=23, N = 10* | *P*=0.018 |  |
| 6c (Early amp, Control vs SR) | Normal | Paired t-test | *het: n = 19, N = 12* | *P=* 7.7x10^-6^ |  |
| 6d (Early amp, Control vs SR) | Normal | Paired t-test | *stg: n = 14, N = 10* | *P=* 3.9x10^-4^ |  |
| 6h (late amp, Control vs strych) | Normal | Paired t-test | *wt: n = 12, N = 5* | *P=* 4.2x10^-3^ |  |
| 6h (Off amp, Control vs strych) | Normal | Paired t-test | *wt: n = 12, N = 5* | *P=* 2.3x10^-2^ |  |
| 7b (het) | Normal | unpaired t-test | *het: n = 28, N=16* | *P*=4.6x10^-3^ |  |
| 7b (stg) | Normal | unpaired t-test (Welch’s correction) | *stg: n=24, N=11* | *P*=3.7x10^-4^ |  |
| 8c (amp fast) | Normal | unpaired t-test | *het: n = 5, N = 4*  *stg: n=5, N = 3* | *P*=0.523 | 0.1033 |
| 8c (amp slow) | Normal | unpaired t-test (Welch’s correction) | *het: n = 5, N = 4*  *stg: n=5, N = 3* | *P*=0.086 | 0.0954 |
| 8c (t fast) | Normal | unpaired t-test | *het: n = 5, N = 4*  *stg: n=5, N = 3* | *P*=0.012 | 0.1077 |
| 8c (t slow) | Normal | unpaired t-test | *het: n = 5, N = 4*  *stg: n=5, N = 3* | *P*=0.452 | 0.1077 |
| 9c (Off DSI) | Normal | unpaired t-test | *het: n = 43, N = 28*  *stg: n=20, N = 9* | *P*=1.81x10^-3^ | 0.4437 |
| 9c (On DSI) | Normal | unpaired t-test | *het: n = 43, N = 28*  *stg: n=20, N = 9* | *P*=0.384 |  |
| 9d (Off Amp) | Normal | unpaired t-test | *het: n = 43, N = 28*  *stg: n=20, N = 9* | *P*=0.028 |  |
| 9d (On Amp) | Normal | unpaired t-test | *het: n = 43, N = 28*  *stg: n=20, N = 9* | *P*=0.192 | 0.4437 |
| 9 (On-Off angle) | Non-normal | Mann-Whitney | *het: n = 43, N = 28*  *stg: n=20, N = 9* | *P*=5.26x10^-3^ |  |
| 11b (narrow bar) | Normal | Repeated measures two-way ANOVA (Sidak’s post hoc comparisons) | *het: n = 27, N = 16;*  *stg: n=18, N = 6* | Genotype *P*=0.0086  Speed *P*<0.0001  Interaction *P*=0.7433 |  |
| 11b (wide bar) | Normal | Repeated measures two-way ANOVA (Sidak’s post hoc comparisons) | *het: n = 27, N = 16;*  *stg: n=18, N = 6* | Genotype  *P*=0.1685  Speed  *P*<0.0001  Interaction *P*=0.9031 |  |
| 11e (ctrl) | Normal | Repeated measures two-way ANOVA (Sidak’s post hoc comparisons) | *het: n = 27, N = 16;*  *stg: n=18, N = 6* | Bar Width *P*=0.0526  Speed *P*<0.0001  Interaction *P*=<0.0001 |  |
| 11e (*stg*) | Normal | Repeated measures two-way ANOVA (Sidak’s post hoc comparisons) | *het: n = 27, N = 16;*  *stg: n=18, N = 6* | Bar Width *P*=0.9402  Speed *P*<0.0001  Interaction *P*=<0.03 |  |
