# Supplementary material for: TARPγ2 Is Required for Normal AMPA Receptor Expression and Function in Direction-Selective Circuits of the Mammalian Retina
Source: eNeuro. 2023 Aug 10;10(8):ENEURO.0158-23.2023. doi: 10.1523/ENEURO.0158-23.2023 (PMC10431237; doi:10.1523/ENEURO.0158-23.2023)
Supplement: Extended Data Table 2-1 — Summary of statistical tests used in this study. Download Table 2, DOC file [file enu-eN-NWR-0158-23-s03.doc]

**Extended Data Table 2-1. Details of antibodies used in this study**

| **Antibody** | **Host** | **Source** | **Cat#** | **Dilution** | **Immunizing antigen** | **Validation** |
| --- | --- | --- | --- | --- | --- | --- |
| TARPɣ2  RRID:AB_10675452 | mouse | Neuromab | 73-242 | 1:8 | Fusion protein amino acids 203-323 (cytoplasmic C-terminus) of rat TARPGamma2 | Knockout validated - this study and by western blot (see datasheet) |
| TARPɣ2/4/8  RRID:AB_10676103 | mouse | Neuromab | 75-252 | 1:100 | Fusion protein amino acids 203-323 (cytoplasmic C-terminus) of rat TARPGamma2 | Knockout validated in this study. Also validated in immunoblots of extracts of COS cells transiently transfected with GFP tagged TARPGamma2/3/4/8  (see datasheet) |
| GluA2  RRID:AB_10674575 | mouse | Neuromab | 73-002 | 1:40 | Fusion protein amino acids 834-883. cytoplasmic C-terminus) of rat GluA2/GluR2 | Validated for immunofluorescence, immunoblot and IHC in brain. KO validated (see datasheet) |
| GluR1  RRID:AB_2113602 | rabbit | EMD Millipore | #AB1504 | 1:50 | KLH-conjugated linear peptide corresponding to human Glutamate receptor 1 at the cytoplasmic domain | Evaluated by western blot in mouse brain lysate. See extensive literature linked to RRID. |
| GluR4 RRID:AB_90711 | rabbit | EMD Millipore | #AB1508 | 1:200 | Peptide mapping at the C-terminus (RQSSGLAVIAS) of GluA4 | Western blots of brain showing a single band comigrating with GluR4 expressed in transfected cells (manufacturer technical information) Pattern as described previously in retina (Haverkamp et al., 2000, 2001; Grunert et al., 2002) |
| GluA3 RRID:AB_2113895 | goat | Santa Cruz Biotech | SC-7612 | 1:1000 | Peptide mapping at the C-terminus of human GluA3 | Knockout validated in mouse cochlea. May show very low-level cross reactivity with GluA2 due to sequence homology (Rutherford et al., 2022, Elife). |
| Calretinin  RRID:AB_2721226 | rabbit | Swant | #7697 | 1:100,000 | recombinant human calretinin containing a 6-his tag at the N-terminal | Band of expected molecular weight in whole brains of different species including mouse and macaque (see datasheet). |
| GABA_A_R b2, b3 subunit  RRID:AB_2109419 | mouse | EMD Millipore | MAB341 | 1:400 | Extracellular domain of the beta2 & beta3 subunits of the GABAA receptor | Validated by western blot of mouse brain membrane lysates (see datasheet) |
| PSD95 (Clone K28/43)  RRID:AB_10698024 | mouse | Neuromab | 73-028 | 1:200 | Fusion protein amino acids 77-299 (PDZ domains 1 and 2) of human PSD-95 | Knockout validated in western blots of mouse hippocampal membranes (see datasheet) |
| Choline acetyltransferase (ChAT)  RRID:AB_90650 | goat | Millipore | #AB144 | 1:500 | Human placental enzyme | Pattern of staining as established for SACs in prior studies (e.g. Li et al., 2016). See also RRID. |
